# A Dye‐Sensitized Sensor for Oxygen Detection under Visible Light

**DOI:** 10.1002/advs.202405694

**Published:** 2024-08-13

**Authors:** Lionel Wettstein, Julia Specht, Vera Kesselring, Leif Sieben, Yanlin Pan, Daniel Käch, Dominika Baster, Frank Krumeich, Mario El Kazzi, Máté J. Bezdek

**Affiliations:** ^1^ Department of Chemistry and Applied Biosciences ETH Zürich Vladimir‐Prelog‐Weg 1 Zürich 8093 Switzerland; ^2^ PSI Center for Energy and Environmental Sciences Paul Scherrer Institute Forschungsstrasse 111 Villigen CH‐5232 Switzerland

**Keywords:** carbon nanotubes, chemiresistive sensors, oxygen, photosensitizers, rhenium complex

## Abstract

Sensors that can accurately assess oxygen (O_2_) concentrations in real time are crucial for a wide range of applications spanning personal health monitoring, environmental protection, and industrial process development. Here a high‐performance chemiresistive sensor that allows for the rapid detection of O_2_ at room temperature under visible light illumination is described. Inspired by the operating principles of dye‐sensitized solar cells, the chemiresistor is based on a single‐walled carbon nanotube‐titania hybrid (SWCNT‐TiO_2_) bearing a molecular Re‐based photosensitizer [(^P^bpy)(CO)_3_ReBr] (^P^bpy = 4,4′‐[P(O)(OH)_2_]_2_‐2,2′‐bipyridine). The resulting **SWCNT‐TiO_2_‐Re** composite undergoes photoinduced charge transfer that is sensitive to ppb levels of O_2_, thereby yielding a rapid and reversible chemiresistive response. Owing to its unique mode of operation and robust components, the sensor shows a high degree of selectivity for O_2_ over a range of interferants, humidity tolerance, and multimonth benchtop stability. The approach presented herein demonstrates the translatability of concepts in light harvesting to the development of robust, rapid, and low‐power sensing technologies.

## Graphical abstract

The accurate detection of oxygen (O_2_) is of paramount importance in a wide range of applications spanning the biological, medical, and environmental sciences. Inspired by concepts in solar light harvesting, a high‐performance dye‐sensitized sensor is developed that can selectively detect O_2_ under visible light illumination.

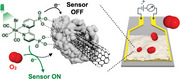

## 1 Introduction

Dioxygen (O_2_) is ubiquitous in the environment and is essential for life.^[^ ^1^ ^]^ As such, the accurate measurement of O_2_ concentration is crucial for a range of applications spanning the medical, biological, and environmental sciences including water quality monitoring^[^ ^2^ ^]^ and evaluating cell health or disease progression.^[^ ^3^, ^4^ ^]^ Information about O_2_ levels is equally important in industrial settings, as exemplified by the central role of O_2_ detectors in personnel health protection,^[^ ^5^ ^]^ automobile exhaust analysis^[^ ^6^ ^]^ and process control in the food and pharmaceutical sectors.^[^ ^7^, ^8^ ^]^ To meet the constantly evolving operational demands of O_2_ detection in such a diverse array of environments, the discovery of new materials and O_2_ sensing mechanisms is critical.^[^ ^9^, ^10^ ^]^

State‐of‐the‐art oxygen sensing is typically accomplished by colorimetric and luminescent probes,^[^ ^11^, ^12^, ^13^ ^]^ electrochemistry,^[^ ^14^, ^15^, ^16^, ^17^ ^]^ or using metal oxide‐containing resistive sensors.^[^ ^6^, ^9^, ^18^, ^19^, ^20^, ^21^ ^]^ Although such approaches can provide sensitive means of O_2_ detection, they are not ideal for applications that require real‐time analysis at ambient conditions using compact, low‐cost, and portable devices with low power requirements.^[^ ^22^ ^]^ For this purpose, lightweight chemiresistors based on carbon nanotubes represent an attractive detection platform owing to room‐temperature operation and a possibility of incorporation into ultra‐small microelectronic devices.^[^ ^23^, ^24^, ^25^ ^]^ While both single‐walled carbon nanotubes (SWCNTs) and multi‐walled carbon nanotubes (MWCNTs) can be utilized for sensing applications, SWCNTs typically offer enhanced sensitivities given that the entirety of the nanotube is available for analyte interactions.^[^ ^23^ ^]^ By contrast, only the outermost graphene surface is in contact with the environment in MWCNTs, which restricts the diversity of mechanisms that can give rise to a sensing signal. Accordingly, SWCNTs are known to be sensitive to O_2_, although this response is nonspecific as π‐sidewalls are indiscriminate adsorption sites for a variety of analytes.^[^ ^26^, ^27^, ^28^ ^]^ The development and incorporation of a recognition element (i.e., a “selector”) is hence necessary with SWCNTs in order to translate an interaction with O_2_ into a sensitive yet selective sensing response.

**Figure 1:**
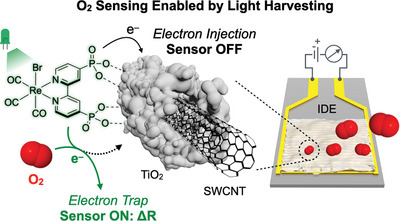
The O_2_ sensing concept reported in this study, involving photoinduced charge transfer from a molecular Re‐based sensitizer to SWCNT‐TiO_2_. Photoexcited electrons are trapped in the presence of O_2_, yielding a sensing signal in the form of a change in device resistance (Δ*R*). SWCNT = single‐walled carbon nanotube, IDE = interdigitated electrode.

Given the exceptional sensitivity of photoexcited states to the presence of O_2_,^[^ ^29^ ^]^ in this work we pursue an O_2_ detection strategy with SWCNTs bearing a selector that can harvest visible light. This approach is reminiscent of excited‐state quenching in luminescent O_2_ probes^[^ ^13^ ^]^ with the key advantage that the sensing output is a resistance change in the underlying SWCNT network that can be measured without the need for bulky and expensive analytical instrumentation. To achieve long‐term device stability under photochemical conditions, we drew inspiration from the design of dye‐sensitized solar cells (DSSCs), where visible light harvesting is accomplished by a sensitizer molecule immobilized on a metal oxide surface.^[^ ^30^, ^31^, ^32^, ^33^ ^]^ Specifically, we reasoned that encapsulation of conductive SWCNTs with a metal oxide bearing a molecular light harvester should be an effective strategy to obtain an O_2_‐sensitive, yet robust chemiresistor with long‐term operational photostability (**Figure** **1**). This approach turns the known detrimental effect of O_2_ on DSSC performance into an advantage for sensing applications and addresses the limitations of existing carbon nanomaterial‐based O_2_ sensors that include SWCNTs decorated with surface functionalities,^[^ ^34^ ^]^ nanoparticles,^[^ ^35^ ^]^ 2D materials,^[^ ^36^ ^]^ and inorganic compounds.^[^ ^37^, ^38^ ^]^ While valuable for correlating material structure with sensory function, each of these reported methods features some combination of humidity intolerance, low sensitivity, narrow selectivity, or short device lifetime that hamper their widespread application.

In this work, we describe a dye‐sensitized chemiresistor that operates at room temperature and can selectively detect ppb levels of O_2_ using visible light. Our device is based on a SWCNT‐TiO_2_ composite bearing the robust molecular photosensitizer^[^ ^39^, ^40^ ^]^ [(^P^bpy)(CO)_3_ReBr] (^P^bpy = 4,4′‐[P(O)(OH)_2_]_2_‐2,2′‐bipyridine; **SWCNT‐TiO_2_‐Re**). In analogy to the operating principles of DSSCs,^[^ ^41^ ^]^ we show that [(^P^bpy)(CO)_3_ReBr] is capable of harvesting visible light, thereby injecting electrons into the underlying SWCNT‐TiO_2_ network. The presence of O_2_ alters this charge injection process and leads to a resistance change in the material that forms the basis of the sensor response (Figure 1). We demonstrate that **SWCNT‐TiO_2_‐Re** is an exceptionally robust chemiresistor and is capable of the rapid, sensitive, and selective detection of O_2_ under a diverse range of environmental conditions.

## 2 Results and Discussion

**Figure 2:**
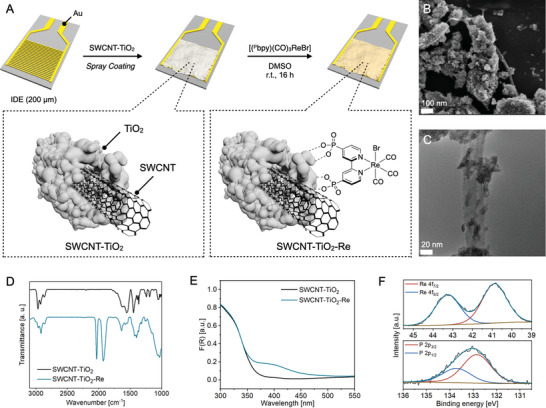
A) Stepwise device fabrication by spray coating of SWCNT‐TiO_2_ onto the Au IDE pattern followed by sensitizer immobilization by soaking in [(^P^bpy)(CO)_3_ReBr] solution to yield **SWCNT‐TiO_2_‐Re**. B) SEM image of **SWCNT‐TiO_2_‐Re** (U_acc_ = 2 kV). C) TEM image of **SWCNT‐TiO_2_‐Re** showing TiO_2_ particles attached to SWCNT bundles (U_acc_ = 80 kV). D) IR spectra (KBr) of SWCNT‐TiO_2_ and **SWCNT‐TiO_2_‐Re**. E) UV‐Vis DRS spectra of SWCNT‐TiO_2_ and **SWCNT‐TiO_2_‐Re**. F(R) = Kubelka–Munk function. F) Re 4f (top) and P 2p (bottom) XPS spectra of **SWCNT‐TiO_2_‐Re**.

We commenced our studies with the deposition of uniformly dispersed SWCNT‐TiO_2_ films on interdigitated Au electrode patterns by spray coating (**Figure** **2**A).^[^ ^42^ ^]^ Throughout this work, we utilized electrode patterns having 200 µm gaps wherein the spray coating procedure was optimized to obtain films with 1–5 kΩ resistance. Following SWCNT‐TiO_2_ deposition, soaking the device in a DMSO solution (2.0 × 10^−3^ m) of the sensitizer [(^P^bpy)(CO)_3_ReBr] for 16 h at room temperature achieved the immobilization of the metal complex to yield the targeted chemiresistor **SWCNT‐TiO_2_‐Re**. Similar to the morphology of SWCNT‐TiO_2_,^[^ ^42^ ^]^ in **SWCNT‐TiO_2_‐Re**, transmission electron microscopy (TEM) and scanning electron microscopy (SEM) revealed the attachment of amorphous TiO_2_ particles (*d* = 296 ± 94 nm) to SWCNT bundles (Figure 2B,C and Figures S3–S8, Supporting Information). The atomic composition of **SWCNT‐TiO_2_‐Re** was further confirmed by energy‐dispersive X‐ray spectroscopy (EDX) as shown in Figures S4 and S5 (Supporting Information).

To probe the composition and surface speciation of **SWCNT‐TiO_2_‐Re** in greater detail, the film was subjected to infrared, ultraviolet‐visible diffuse reflectance, and X‐ray photoelectron spectroscopic analysis (IR, UV‐Vis DRS, XPS, respectively). The solid‐state IR spectrum of **SWCNT‐TiO_2_‐Re** (KBr pellet) showed diagnostic vibrations at 2033 cm^–1^ and 1928 cm^–1^ assignable to the carbonyl ligands in TiO_2_‐bound [(^P^bpy)(CO)_3_ReBr] (Figure 2D).^[^ ^43^ ^]^ In addition, UV‐Vis DRS analysis revealed a feature in the spectrum of **SWCNT‐TiO_2_‐Re** with an onset of 484 nm, which is consistent with the solution‐state UV‐Vis absorption spectrum of [(^P^bpy)(CO)_3_ReBr] (Figure 2E). Besides the expected features for carbon, titanium and oxygen, XPS analysis revealed peaks corresponding to Re 4f_5/2_ and Re 4f_7/2_ binding energies at 43.29 and 40.94 eV, respectively, supporting the incorporation of Re in **SWCNT‐TiO_2_‐Re** (Figure 2F).^[^ ^43^ ^]^ High resolution P 2p, N 1s, and Br 3d scans were also collected and indicated the presence of phosphonate, coordinated bipyridine, and bromide moieties, respectively (Figure 2F and Figures S13 and S14, Supporting Information). Overall, IR, UV‐Vis DRS, and XPS data support the successful surface‐immobilization of [(^P^bpy)(CO)_3_ReBr] in **SWCNT‐TiO_2_‐Re**.

**Figure 3:**
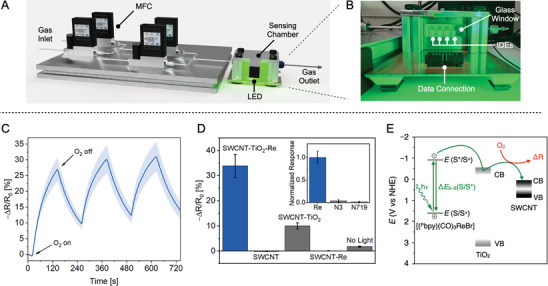
A) CAD (computer‐aided design) drawing of the sensor testing platform. MFC = mass flow controller, LED = light emitting diode. B) Photograph of the sensing chamber housing four parallel IDE devices under green light illumination. C) Averaged resistance trace of **SWCNT‐TiO_2_‐Re** in response to three repeated 2 min O_2_ exposures (1000 ppm). D) Sensing response of **SWCNT‐TiO_2_‐Re** to a 2 min O_2_ exposure (1000 ppm) compared to control devices with systematically omitted components. Inset: comparison of the normalized device response to O_2_ upon varying the photosensitizer component in **SWCNT‐TiO_2_‐Re**. Shaded areas and error bars represent standard deviations (*N* = 4 chemiresistors); all data were collected under green light irradiation at room temperature using dry nitrogen carrier gas. E) Simplified relative alignment of SWCNT, TiO_2_, and [(^P^bpy)(CO)_3_ReBr] electronic bands in **SWCNT‐TiO_2_‐Re** and proposed mechanism of O_2_ detection. Values for TiO_2_ and SWCNTs were adapted from ref. [45], while energy levels for [(^P^bpy)(CO)_3_ReBr] were experimentally determined (see Supporting Information for details). VB = valence band, CB = conduction band.

With the composition and electronic absorption properties of **SWCNT‐TiO_2_‐Re** established, its chemiresistive response was examined under visible light irradiation in the presence and absence of O_2_. We conducted our proof‐of‐concept sensing experiments using a custom‐made setup wherein various amounts of O_2_ (in N_2_) were delivered to a gas‐tight enclosure housing four devices at a flow rate of 1 L min^−1^ (**Figure** **3**A). Our enclosure featured a glass window that allowed for controlled visible light irradiation of the sensors without disturbance of the gas flow (Figure 3B). Given the electronic absorption characteristics of **SWCNT‐TiO_2_‐Re** (see above), green light was selected for the photosensing experiments (*λ* = 516 nm, ≈200 mW mm^−2^). Accordingly, exposure of **SWCNT‐TiO_2_‐Re** to 0.1% (1000 ppm) of O_2_ for 2 minutes at room temperature under green light resulted in a significant sensor response, taken as the normalized change in device resistance [Δ*R*/*R*_0_ (%) = (*R* − *R*_0_)/*R*_0_ × 100%; *R*_0_ = initial resistance] of −30.5 ± 4.3% (Figure 3C). The sensor response magnitude was found to be consistent after repeated exposures and was reversible. The maximum rate of resistance change was observed after 6 seconds of O_2_ exposure, and baseline recovery was achieved after 9 minutes of purging with N_2_ (Figures S19 and S20, Supporting Information). **SWCNT‐TiO_2_‐Re** is hence viable for rapid and reversible O_2_ detection at room temperature under green light illumination, motivating further experiments to examine the origin of the chemiresistive response.

Control devices were fabricated to determine whether all sensor components are needed for the observed O_2_ response (Figure 3D and Figure S25, Supporting Information). Devices containing only SWCNTs exhibited a negligible O_2_ response, as did those devices fabricated with the exclusion of TiO_2_ (SWCNT‐Re). Further, omission of SWCNTs yielded a composite (TiO_2_‐Re) with resistance values too high to be monitored using our data acquisition unit. Omission of [(^P^bpy)(CO)_3_ReBr] led to a significantly smaller and, importantly, an irreversible signal with SWCNT‐TiO_2_. Such signal irreversibility may point to the filling of TiO_2_ surface oxygen vacancies (defects) upon exposure of SWCNT‐TiO_2_ to O_2_.^[^ ^44^ ^]^ Interestingly, substituting [(^P^bpy)(CO)_3_ReBr] for other sensitizers commonly applied in DSSCs such as the ruthenium dyes^[^ ^32^ ^]^ [(^C1^bpy)_2_Ru(NCS)_2_] (^C1^bpy = 4,4′‐(C(O)OH)_2_‐2,2′‐bipyridine; **N3**) or [(*n*‐Bu)_4_N]_2_[(^C2^bpy)_2_Ru(NCS)_2_] (^C2^bpy = 4‐(C(O)OH)−4′‐(C(O)O)−2,2′‐bipyridine; **N719**) had a detrimental effect on the O_2_ sensing performance (Figure 3D inset and Figure S26, Supporting Information). Finally, exposing **SWCNT‐TiO_2_‐Re** to O_2_ in the dark did not produce a significant sensing response. Overall, our control experiments establish that SWCNTs, TiO_2_, the [(^P^bpy)(CO)_3_ReBr] sensitizer, and green light are all crucial for the highly sensitive and reversible chemiresistive O_2_ response of **SWCNT‐TiO_2_‐Re**.

We were interested in corroborating our initial hypothesis for the origin of the O_2_ sensing response in **SWCNT‐TiO_2_‐Re** under visible light. Our understanding of the system is guided by previous studies on SWCNT‐TiO_2_ under UV light irradiation wherein photo‐induced electron injection was proposed to occur from TiO_2_ to the SWCNTs.^[^ ^42^, ^45^ ^]^ Given that semiconducting SWCNTs exhibit p‐type behavior under ambient conditions,^[^ ^46^ ^]^ electron injection from TiO_2_ results in a reduction of carrier (hole) density in SWCNTs, thus producing an increased device resistance. While high‐energy UV light is necessary to efficiently trigger this process in SWCNT‐TiO_2_, we hypothesized that introduction of [(^P^bpy)(CO)_3_ReBr] should sensitize TiO_2_ and enable electron injection under visible light irradiation (Figure 3E).^[^ ^47^, ^48^ ^]^ Indeed, the relevant electronic absorption band of [(^P^bpy)(CO)_3_ReBr] is known to bear principally metal‐to‐ligand charge‐transfer (MLCT) character, dominated by a transition from Re‐centered orbitals to the TiO_2_‐bound bipyridyl π* orbital which facilitates electron injection.^[^ ^49^, ^50^, ^51^ ^]^ Further, [(^P^bpy)(CO)_3_ReBr] exhibits an excitation energy [Δ*E*_0–0_(S/S*)] of 2.56 eV and an excited‐state potential [*E*(S*/S^+^)] of −0.93 V (vs NHE),^[^ ^52^ ^]^ and is thus energetically well‐positioned for green light‐induced electron injection into SWCNTs via the TiO_2_ conduction band (Figure 3E). Given that [(^P^bpy)(CO)_3_ReBr] features a ground‐state potential [*E*(S/S^+^)] of ≈1.63 V, we expect any photo‐generated Re(II) species to be capable of reacting with surface H_2_O (*E*_ox_ = 1.23 V)^[^ ^53^ ^]^ to extract electrons and induce recovery of baseline resistance. Consistent with this view, exposure of **SWCNT‐TiO_2_‐Re** to green light under N_2_ resulted in a sharp increase in device resistance, which was reversible when the light was switched off. By contrast, SWCNT‐TiO_2_ exhibited a significantly smaller change in device resistance under green light, likely due to its larger band gap (≈3.5 eV) that leads to inefficient green light harvesting (Figure S28, Supporting Information). As described above, exposure of **SWCNT‐TiO_2_‐Re** to O_2_ under green light illumination resulted in a reversible resistance drop, an effect that was more pronounced with increasing light irradiance (Figure S29, Supporting Information). Taken together, these results suggest that the presence of O_2_ hinders photoinduced electron injection, likely by trapping photoexcited electrons in **SWCNT‐TiO_2_‐Re**,^[^ ^54^, ^55^, ^56^ ^]^ and hence gives rise to a sensing signal through a reversible decrease in device resistance.

**Figure 4:**
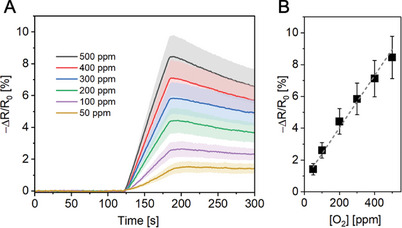
A) Averaged resistance trace of **SWCNT‐TiO_2_‐Re** in response to a 1 min O_2_ exposure of various concentrations (50–500 ppm). B) Sensing response of **SWCNT‐TiO_2_‐Re** to a 1 min O_2_ exposure of various concentrations (50–500 ppm). Shaded areas and error bars represent standard deviations (*N* = 4 chemiresistors); all data were collected under green light irradiation at room temperature using dry nitrogen carrier gas.

With insights in hand concerning the origin of the O_2_ sensing response in **SWCNT‐TiO_2_‐Re,** we proceeded to evaluate the comprehensive sensing performance metrics of our devices. Exposing **SWCNT‐TiO_2_‐Re** to various O_2_ concentrations resulted in a linear sensing response for the range 50–500 ppm O_2_, enabling us to calculate a limit of detection (LOD) of 2.2 ppm for a 1‐minute analyte exposure (**Figure** **4**A,B). Extending the exposure time to 60 min in turn lowered the detection limit to 157 ppb (Figure S30, Supporting Information). Exposure of **SWCNT‐TiO_2_‐Re** to higher O_2_ amounts (up to 20%) yielded a sensor response that was exponentially proportional to O_2_ concentration (Figure S31, Supporting Information). Furthermore, stable O_2_ sensing performance was observed in the relative humidity (R.H.) range of 0–80% (Figure S32, Supporting Information). These results are particularly remarkable considering that humidity interference is among the most significant challenges hindering the adoption of SWCNT‐based chemiresistors in real‐world applications.^[^ ^57^ ^]^ Overall, our results establish that **SWCNT‐TiO_2_‐Re** is a highly sensitive O_2_ detector, capable of operating under environmentally relevant conditions.

**Figure 5:**
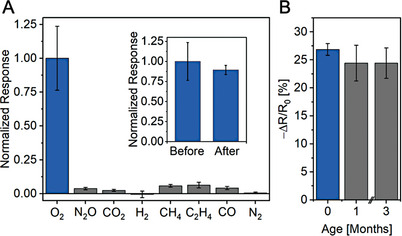
A) Normalized sensing response of **SWCNT‐TiO_2_‐Re** to a 1 min exposure (1000 ppm) of various gases in comparison with the sensor response to O_2_. Inset: comparison of the device response to O_2_ before/after interferant exposure. B) Sensing response of **SWCNT‐TiO_2_‐Re** to a 1 min O_2_ exposure (1000 ppm) after storage on a laboratory bench for varying time periods. Error bars represent standard deviations (*N* = 4 chemiresistors); all data were collected under green light irradiation at room temperature using dry nitrogen carrier gas.

With sensitivity metrics established for **SWCNT‐TiO_2_‐Re**, its selectivity with respect to other potential interferant gases was examined. Shown in **Figure** **5**A, **SWCNT‐TiO_2_‐Re** was found to exhibit clear selectivity for O_2_ over oxidizing and reducing gases such as nitrous oxide (N_2_O), carbon dioxide (CO_2_), hydrogen (H_2_) and methane (CH_4_). Besides establishing a key performance metric, the results of our selectivity studies also broaden our mechanistic understanding of the system. In particular, the lack of a significant response to CO_2_ indicates that light irradiation likely does not produce anionic [(^P^bpy)(CO)_3_ReBr]^–^ species at the sensor surface, which are well‐known to react with CO_2_.^[^ ^58^, ^59^ ^]^ Further, the sensor's insensitivity toward H_2_ suggests that, unlike metal oxide sensors that operate at elevated temperatures,^[^ ^60^, ^61^ ^]^ **SWCNT‐TiO_2_‐Re** does not undergo reduction by H_2_ and hence does not produce a response.

Besides oxidizing and reducing gases, we also exposed our devices to ethylene (C_2_H_4_) and carbon monoxide (CO), both of which can react with transition metal‐based receptors and trigger a response.^[^ ^62^, ^63^, ^64^ ^]^ The lack of a significant response to either gas argues against photoinduced ligand dissociation at [(^P^bpy)(CO)_3_ReBr] during sensing, which would open a coordination site where C_2_H_4_ or CO could bind. Importantly, we tested the response of **SWCNT‐TiO_2_‐Re** to O_2_ following the exposure to the interferant gases and found no signal attenuation, ruling out selector poisoning during our selectivity studies (Figure 5A, inset). Further, stability tests showed no significant loss of O_2_ sensing response neither after storage of **SWCNT‐TiO_2_‐Re** for 3 months under ambient conditions nor after extended O_2_ exposures under light (Figure 5B and Figures S34 and S35, Supporting Information). Collectively, these studies emphasize that **SWCNT‐TiO_2_‐Re** demonstrates exceptional robustness and selectivity, outperforming state‐of‐the‐art chemiresistive O_2_ gas sensing technologies (see Table S2 in the Supporting Information).

**Figure 6:**
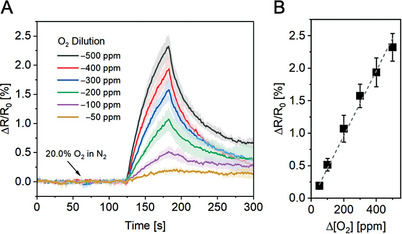
A) Averaged resistance traces of **SWCNT‐TiO_2_‐Re** in response to 1 min decreases of O_2_ concentration ([O_2_]_0_ = 20.0%; ΔO_2_ = 50–500 ppm). B) Sensing response of **SWCNT‐TiO_2_‐Re** to 1 min decreases of O_2_ concentration (ΔO_2_ = 50–500 ppm). Shaded areas and error bars represent standard deviations (*N* = 4 chemiresistors); all data were collected under green light irradiation at room temperature using dry synthetic air carrier gas.

Having established the utility of **SWCNT‐TiO_2_‐Re** for trace O_2_ detection, we explored its suitability for portable field deployment by investigating whether the device maintains sensitivity to O_2_ concentration changes even when operated under ambient oxygen levels. For this experiment, we equilibrated the chemiresistor under green light irradiation using synthetic air (20.0% O_2_ in N_2_) as carrier gas and systematically decreased the concentration of O_2_ by diluting the gas mixture with N_2_, thus triggering an increase in device resistance (**Figure** **6**A). Remarkably, the device maintained high sensitivity to O_2_ concentration changes with as low as 26 ppm differences being detectable after 1 minute of O_2_ dilution. Shown in Figure 6B, the rapid and reversible sensor response varied linearly with O_2_ concentration. This result underscores the high dynamic range of the sensing composite under green light illumination (0–200 000 ppm) and unlocks the possibility of using the device in applications that require exceptional O_2_ sensitivity under ambient conditions.

## 3 Conclusion

In summary, we have developed a chemiresistive O_2_ sensor fabricated from a single‐walled carbon nanotube‐TiO_2_ composite incorporating a molecular rhenium‐based sensitizer. Inspired by the dye‐sensitization of TiO_2_ for solar cell applications, we conceived and constructed a sensor that operates under visible light illumination at room temperature. The device offers ppb‐level sensitivity to O_2_ with rapid response times and multi‐month stability. Due to its unique mode of operation, the chemiresistor exhibits exquisite selectivity for O_2_ over an array of interferant gases and shows robust performance under a wide range of O_2_ concentrations and humidity levels. These investigations demonstrate that concepts in light harvesting can be translated to achieve high‐performance O_2_ detection and should inspire new sensing strategies in industrial settings as well as across the biological, medical, and environmental sciences.

## Conflict of Interest

The authors declare the following competing financial interest(s): A patent has been filed on this technology.

## Supporting information

Supporting Information

## Data Availability

The data that support the findings of this study are available in the supplementary material of this article.
