# Supplementary material for: A Dye‐Sensitized Sensor for Oxygen Detection under Visible Light
Source: Adv Sci (Weinh). 2024 Aug 13;11(43):2405694. doi: 10.1002/advs.202405694 (PMC11578297; doi:10.1002/advs.202405694)
Supplement: Supplementary file 1 — Supporting Information [file ADVS-11-2405694-s001.pdf]

ADVANCED SCIENCE
Open Access

Supporting Information

for *Adv. Sci.*, DOI 10.1002/advs.202405694

A Dye-Sensitized Sensor for Oxygen Detection under Visible Light

*Lionel Wettstein, Julia Specht, Vera Kesselring, Leif Sieben, Yanlin Pan, Daniel Käch, Dominika Baster, Frank Krumeich, Mario El Kazzi and Máté J. Bezdek**

Supporting Information

A Dye-Sensitized Sensor for Oxygen Detection under Visible Light

Lionel Wettstein,[1] Julia Specht,[1] Vera Kesselring,[1] Leif Sieben,[1] Yanlin Pan,[1] Daniel Käch,[1] Dominika Baster,[2] Frank Krumeich,[1] Mario El Kazzi,[2] and Máté J. Bezdek*[1]

[1]Department of Chemistry and Applied Bioscience

ETH Zürich

Vladimir-Prelog-Weg 1, 8093 Zürich (Switzerland)

*E-mail: mbezdek@ethz.ch

[2]PSI Center for Energy and Environmental Sciences

Paul Scherrer Institute

Forschungsstrasse 111, CH-5232

Table of Contents

I.	General Information	3
II.	Preparation of SWCNT-TiO_2-Re	5
III.	Additional Characterization Data	7
IV.	O_2 Detection Measurements	19
V.	Additional Sensing Data	21
VI.	O_2 Chemiresistor Comparison Table	31
VII.	Energy Levels of [(^{P}bpy)$(CO)_3$ReBr]	32
VIII.	Synthetic Protocols	34
IX.	References	38

I. General Information

Materials: Unless otherwise stated, reagents were used as supplied from commercial sources without any further purification. $Ti(O^iPr)_4$ (99.999% trace metal basis) and $[Re(CO)_5Br]$ were purchased from Sigma-Aldrich and stored in an argon-filled glovebox at −35 °C. SWCNTs (P3-SWNT, > 90% carbonaceous purity, 5–7 wt% metal content, 500 nm–1.5 μm bundle length, 4–5 nm bundle diameter, 1.55 ± 0.1 nm individual tube diameter, lot # 03-A036) were purchased from Carbon Solutions, Inc and dried under high vacuum before storage in an argon-filled glovebox. Triethylamine (Et_3N), $[Pd(PPh_3)_4]$, and indium-tin oxide (ITO) coated glass slides were purchased from Sigma-Aldrich. Triphenylphosphane (PPh_3) was purchased from Apollo. 4,4'-Dibromo-2,2'-bipyridine was purchased from abcr. $[(^Pbpy)(CO)_3ReBr]$ (^{P}bpy = 4,4'-$[P(O)(OH)_2]_2$-2,2'-bipyridine; **Re**) was synthesized by modification of reported procedures (see "Synthetic Protocols" section). The ruthenium dyes $(^Cbpy)_2Ru(NCS)_2$ (^{C}bpy = 4,4'-$(C(O)OH)_2$-2,2'-bipyridine; **N3**) and $[(n\text{-Bu})_4N]_2[(^{C2}bpy)_2Ru(NCS)_2]$ (^{C2}bpy = 4-(C(O)OH)-4'-(C(O)O)-2,2'-bipyridine; **N719**) were purchased from Solaronix and purified by recrystallization. Solvents were dried and de-gassed using an argon-connected MBraun InertGas SPS-7 solvent purification system. The residual water content in solvents was determined using a Mettler-Toledo C30 coulometric Karl Fischer titrator. Following purification, solvents were stored over 3 Å or 4 Å molecular sieves in an argon-filled glovebox. Gases were purchased from Pangas or Air Liquide and equipped with gas-flow regulators: N_2O (5.0 grade), synthetic air (N_2: 80% / O_2: 20%), H_2 (5.0 grade), CO_2 (4.5 grade), CH_4 (5.5 grade), C_2H_4 (3.0 grade), CO (4.7 grade). Interdigitated gold electrodes on ceramic substrate were purchased from DropSens (IDEAU200). All air- and moisture-sensitive manipulations were carried out using standard vacuum line Schlenk technique or in an argon-filled MBraun LabMaster Pro glovebox. Reactions were carried out in oven- or flame-dried glassware equipped with a magnetic stir bar. Microwave synthesis was performed using a Biotage® Initiator+ instrument equipped with Robot Eight.

Cyclic Voltammetry (CV) was performed in an argon-filled MBraun UniLab glovebox with a Gamry Interface 1010E potentiostat/galvanostat/ZRA using a three-electrode electrochemical cell. If not stated otherwise, a $Ag/AgNO_3$ (0.010 M in $[(n\text{-Bu})_4N][PF_6]$ in MeCN, BASi), glassy carbon disk (1.6 mm^2, eDAQ), and Pt-wire were used as reference, working and counter electrodes respectively. The working electrode was polished before each experiment on a pad using an alox-slurry (0.050 mm) and rinsed sequentially with millipore water, isopropanol (^{i}PrOH), and acetone. Experiments were conducted using 0.10 M $[(n\text{-Bu})_4N][PF_6]$ supporting electrolyte in DMF or MeCN. First, a background scan of a blank sample containing electrolyte and solvent was performed to determine the solvent window and ensure a stable potential and the absence of contaminants. Next, the respective compound (1.0 mM) was dissolved in the blank sample and three scans of the full window starting at the open circuit potential (OCP) were recorded. The first scan is reported. CVs were internally referenced against the Fc/Fc^+ couple. If necessary, data smoothening using the Savitzky–Golay method was applied.

Flash column chromatography was performed on a Biotage® Isolera™ One system with Sfär columns using technical grade solvents.

Fluorescence Spectroscopy: Emission spectra were recorded on an Agilent Cary Eclipse Fluorescence Spectrometer.

High-resolution mass spectrometry data was obtained by the mass spectrometry service in the Laboratorium für Organische Chemie at ETH Zürich on a Varian IonSpec Spectrometer for electrospray ionization (ESI). The molecular ions $[M]^+$, $[M+H]^+$, and $[M+Na]^+$ are reported as (m/z).

IR Spectroscopy: FT-IR spectra were measured either in the solid state under air with the ATR technique on a Bruker Tensor or in a glovebox as KBr pellets with a Bruker Alpha II FT-IR spectrometer. KBr pellets were prepared by finely grinding approx. 1 mg of analyte with 100 mg of KBr and pressing the resulting powder into a pellet.

NMR Spectroscopy: Solution NMR measurements were carried out on Bruker Avance 300 MHz, 400 MHz, 500 MHz, and 500 MHz cryoprobe spectrometers. NMR spectra are referenced against residual protonated solvent (^{1}H and ^{13}C), $CFCl_3$ (^{19}F), and H_3PO_4 (^{31}P). Chemical shifts (δ) are reported in parts per million (ppm) and the absolute values of the coupling constants are given in Hertz (Hz). Multiplicities are indicated by s (singlet), d (doublet), t (triplet), q (quartet), m (multiplet), br (broad), and combinations thereof.

Powder X-ray Diffraction (PXRD): PXRD was performed on a Stoe STADIP Dual Setup instrument with Mythen Detectors, using the Cu-Kα1 edge as radiation source (1.540598 Å). SWCNT-TiO_2 was ground to a fine powder and filled into a 0.5 mm Hilgenberg mark tube with 0.01 mm wall thickness for data collection.

Raman Spectroscopy was measured directly on electrodes prepared for photosensing experiments using a LabRam Soleil Raman spectrometer (HORIBA Scientific, France) equipped with a CCD detector and a high-magnification objective lens (100×, 0.9 NA, Nikon, Japan). A laser wavelength of 532 nm was used. Laser power and acquisition time were optimized for each individual measurement.

Scanning Electron Microscopy (SEM): SEM images were obtained on a Zeiss Merlin ultra-high resolution FE-SEM microscope equipped with a Gemini II column and an in-lens and conventional secondary electron detector, using low electron energies of 1 or 2 kV.

(Scanning) Transmission Electron Microscopy (STEM, TEM): TEM and STEM images were obtained from a double corrected JEM-ARM300F (GrandARM JEOL) electron microscope operated at U_{acc} = 80 or 300 kV. The microscope is equipped with (high-angle) annular dark field ((HA)ADF) and bright field (BF) STEM detectors as well as an energy-dispersive X-ray (EDX) spectrometer.

UV-vis Spectroscopy: Solution UV-vis spectra as well as UV-vis diffuse reflectance spectra (DRS) were recorded on an Agilent Technologies Cary5000 UV-vis-NIR spectrophotometer.

X-Ray Photoelectron Spectroscopy (XPS): XPS measurements were carried out with a VG ESCALAB 220iXL spectrometer (Thermo Fisher Scientific) using focused monochromatized Al Kα radiation (1486.6 eV) with a beam size of ≈500 μm^2. The analysis chamber had a recorded residual pressure of approximately 2×10^{-9} mbar. The spectrometer was regularly calibrated using the Ag $3d_{5/2}$ peak with a binding energy of 368.3 eV with a full width at half maximum (FWHM) of 0.78 eV at a pass energy of 30 eV. All survey spectra were recorded with a dwell time of 50 ms, using the pass energy of 50 eV in steps of 0.5 eV. The spectra acquired in a narrow energy scan were recorded with the pass energy of 30 eV in a step size of 0.05 eV. All spectra were calibrated relative to the carbon C 1s peak at 284.7 eV to correct for charging effects. Curve fitting of the core level spectra was performed with the CasaXPS software.[1]

Statistical Analysis: Chemiresistive sensing data was pre-processed by normalization. A drift correction was applied in cases where the absolute response was of interest using linear regression fitting of the baseline. Data of repeated measurements under same conditions are presented as mean ± standard deviation. The sample sizes (*n*) for each statistical analysis are indicated. Statistical analyses were performed using the software OriginPro 2024 (OriginLab Corporation, Massachusetts, USA).

II. Preparation of SWCNT-TiO_2-Re

Fabrication of the SWCNT-TiO_2 Chemiresistor Platform

The SWCNT-TiO_2 hybrid material was prepared according to a modified literature protocol.[2]

Under air, a 20 mL crimp vial equipped with a magnetic stir bar was charged with SWCNT (0.5 mL of 1.0 mg/mL in EtOH, freshly sonicated), dry EtOH (16.5 mL), and deionized H_2O (50 µL).[a] The vial was sealed with a crimp cap, the dispersion was sonicated for 5 minutes, and the reaction vessel was brought into an argon-filled glovebox. Under vigorous stirring, $Ti(O^iPr)_4$ (50 µL) was added dropwise to the dispersion. The reaction mixture was then removed from the glovebox and further stirred for 15 minutes at 1000 rpm. Next, H_2O (3.0 mL) was continuously added to the mixture over the course of 50 minutes using a syringe pump (rate of addition = 0.06 mL/min). The reaction mixture was then further stirred for 15 minutes, and the product was isolated on a nylon membrane filter (0.2 µm), washed with EtOH (3 × 15 mL), and dried under high vacuum.[b] The product (SWCNT-TiO_2) was then removed from the nylon filter and dispersed in deionized H_2O (20 mL) in a crimp vial. This stock dispersion was stored and used to deposit SWCNT-TiO_2 films onto electrodes (Figure S1B).

Prior to each use, the SWCNT-TiO_2 dispersion was sonicated for at least 30 minutes. In a typical procedure, the dispersion was loaded into an airbrush (Revolution BR, Iwata) and manually spray-coated onto the gaps of the interdigitated electrode pattern that was placed on a heating plate set to 150 °C (Figure S1A). Aluminum foil was used to mask the electrode pattern and confine the SWCNT-TiO_2 film deposition to the desired electrode gap region. The dispersion was sprayed intermittently in short (ca. 0.2 sec) bursts at a distance of about 5 cm from the substrate surface. Pressurized air (2 bar) was used as carrier gas. Successful thin film deposition could be observed by momentary wetting of the IDE pattern. Typically, ca. 20 short bursts of the SWCNT-TiO_2 dispersion were sufficient to attain the desired device resistance of 1–5 kΩ. The devices were dried at 180 °C under air overnight.

We noticed that extended storage (4 weeks) of SWCNT-TiO_2 in the aqueous dispersion led to improved photosensing performance of the resulting active sensing material. We attribute this effect to a morphology change due to Ostwald ripening (see SEM section below).

Sensitizer Immobilization on SWCNT-TiO_2

Electrodes bearing the SWCNT-TiO_2 film were soaked in 2.0 mL of a DMSO solution (0.2 mM) of the sensitizer at room temperature in the dark for 16 h (Figure S1C). The devices were then washed by soaking in DMSO (2 x 1 minute) and in deionized water (1 x 10 sec) at room temperature to remove unbound sensitizer, then dried under high vacuum for 90 minutes. When not in use, the electrodes were stored in the dark (Figure S1D).

---

[a] It was found that adding 0.3% H_2O to the reaction mixture was beneficial for the sensing performance of SWCNT-TiO_2-Re.

[b] Typically, around 15 mg of SWCNT-TiO_2 were obtained after drying under high vacuum.

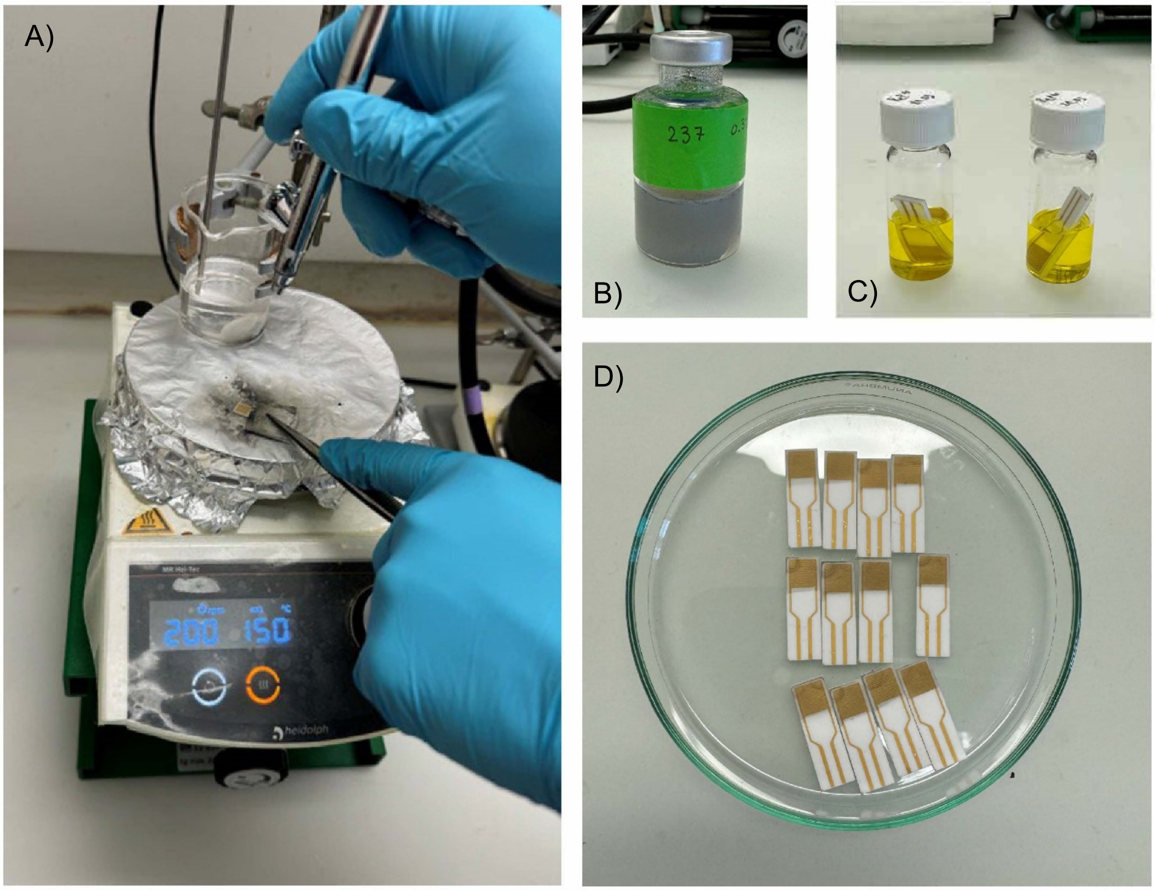

Figure S1. A) Spray-coating setup, where the electrode is masked using aluminium foil (leaving the IDE pattern exposed) and placed on a heating plate at 150 °C. B) SWCNT-TiO_2 dispersion after sonication for 30 minutes. C) Soaking electrodes bearing SWCNT-TiO_2 film in DMSO solution of [(^{P}bpy)$(CO)_3$ReBr]. Each vial contains a pair of electrodes arranged back-to-back to ensure exposure of the SWCNT-TiO_2 film to the surrounding solution. D) Electrodes bearing the title **SWCNT-TiO_2-Re** chemiresistor composite.

III. Additional Characterization Data

TEM and STEM

Sample Preparation

TEM grids for the analysis of SWCNT-TiO_2 were prepared by deposition of a single drop of a dilute aqueous dispersion of SWCNT-TiO_2. For **SWCNT-TiO_2-Re** samples, a DMSO solution of [(^{P}bpy)$(CO)_3$ReBr] (0.3 mL, 2.0 mM) was added dropwise to an aqueous dispersion of SWCNT-TiO_2 (2.0 mL, 0.75 mg/mL) under vigorous stirring. After 1 h, the dispersion was filtered over a Nylon filter (0.2 µm), washed with DMSO (2 × 1.5 mL) and water (1–2 drops), and the filtrate was dried under vacuum for 4 h. The product was then redispersed in water. An aliquot of the dispersion was diluted using EtOH and the TEM sample was prepared by deposition of one drop of this diluted dispersion.

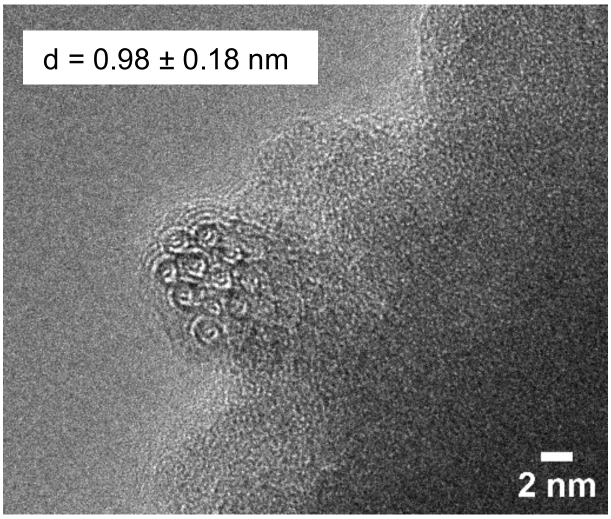

Figure S2. TEM image of a SWCNT bundle cross-section, wherein the image plane is perpendicular to the length of the nanotubes (U_{acc} = 200 kV). Within this bundle, the average SWCNT diameter is 0.98 ± 0.18 nm.

A TEM image depicting a SWCNT bundle cross-section allowed us to calculate an individual tube diameter of 0.98 ± 0.18 nm (Figure S2). TEM images of SWCNT-TiO_2 (not shown) depicted an increase of the average SWCNT bundle diameter from 6.0 ± 2.0 nm to 7.5 ± 4.4 nm in going from 0–4 weeks in a water dispersion, in agreement with SEM results.

STEM measurements were coupled to EDX analysis to evaluate the elemental composition and distribution of the material. Bright-field (BF) and dark-field (DF) images are shown in Figure S3A–C and indicate the presence of $[(^{P}bpy)(CO)_3ReBr]$.

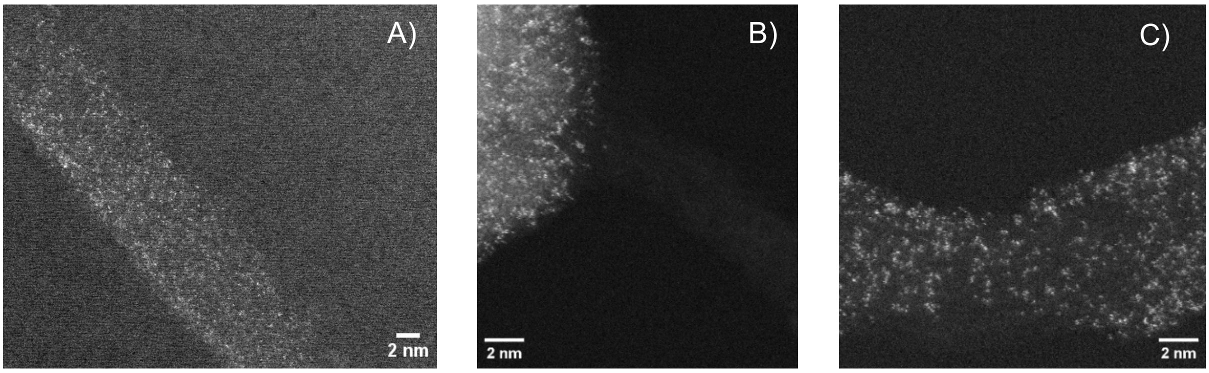

Figure S3. STEM HAADF images of **SWCNT-TiO_2-Re** (JEM-ARM300F, U_{acc} = 300 kV). The bright contrast is due to Re, indicating the presence of $[(^{P}bpy)(CO)_3ReBr]$ on the surface of SWCNTs (A and C) and TiO_2 (B).

Note: The EDX spectrum measured on the SWCNT region (Figure S4, gold) shows the characteristic C K_α edge at 0.277 keV, although this peak is always present and intense due to the supporting amorphous carbon film of the TEM grid. Likewise, the Cu background from the TEM grid is always observed, with its characteristic L_α edge at 0.930 keV, K_α at 8.040 keV, and K_β at 8.910 keV. The EDX spectrum measured on the TiO_2 nanoparticles (Figure S4, blue) clearly indicates the presence of TiO_2, with the Ti L edge at 0.452 keV, and the characteristic K_α at 4.508 keV and K_β at 4.931 keV. The EDX signal for $[(^{P}bpy)(CO)_3ReBr]$ is clearly visible as well, with a Re M edge at 1.842 keV and Re L_α at 8.651 keV. The P K_α at 2.013 keV overlaps with on of the Re M edges, namely at 2.09 keV. Likewise, $[(^{P}bpy)(CO)_3ReBr]$ was found to be present on TiO_2 particles (Figure S5A–C).

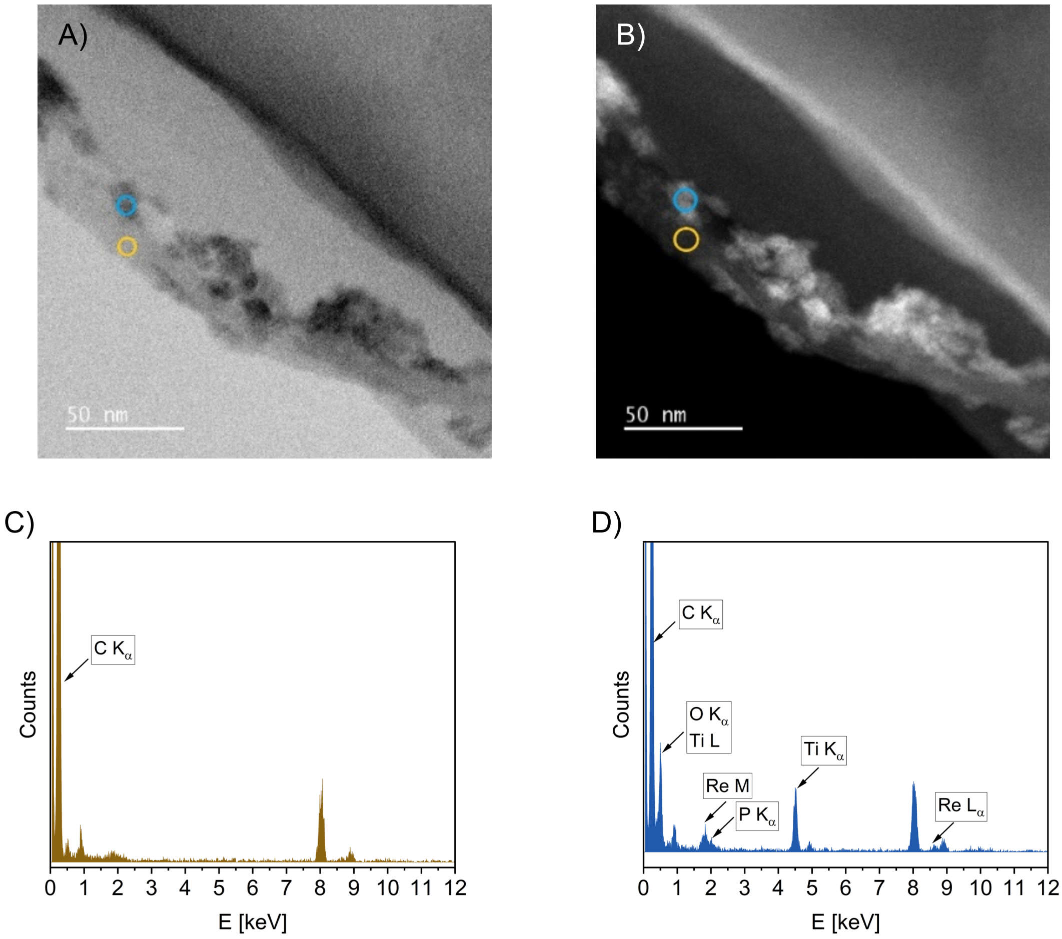

Figure S4. A) STEM BF and B) STEM HAADF images of **SWCNT-TiO$_2$-Re** (JEM-ARM300F, U_{acc} = 80 kV). C) and D) EDX spectra measured at indicated locations on the SWCNT bundle, where the blue spectrum corresponds to the point marked with a blue circle and the gold spectrum to the gold circle.

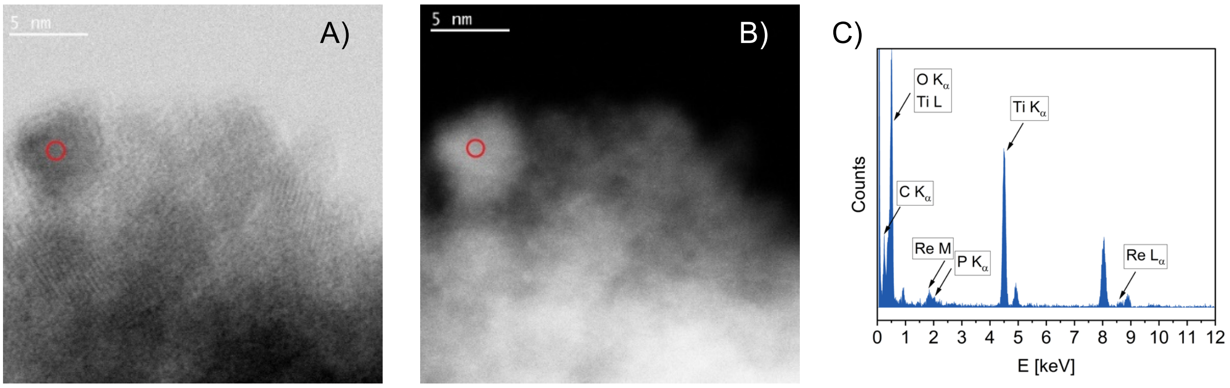

Figure S5. A) STEM BF image and B) STEM DF image of **SWCNT-TiO$_2$-Re** focusing on TiO$_2$ particles (red circle). C) EDX spectrum measured at indicated location.

SEM

Sample Preparation

For SEM measurements, a dispersion of SWCNT-TiO_2 in H_2O was spray-coated onto a silicon wafer at 150 °C. To obtain SEM images of the **SWCNT-TiO_2-Re** composite, a DMSO solution of [(^{P}bpy)$(CO)_3$ReBr] (0.3 mL, 2.0 mM) was added dropwise to an aqueous dispersion of SWCNT-TiO_2 (2.0 mL, 0.75 mg mL^{-1}) under vigorous stirring. After 1 h, the dispersion was filtered over a Nylon filter (0.2 μm), washed with DMSO (2 x 1.5 mL) and water (1–2 drops), and the filtrate was dried under vacuum for 4 h. The product was then redispersed in water and the dispersion was spray-coated on a silicon wafer at 150 °C analogously to the standard electrode preparation. A single burst deposited sufficient material to obtain adequate SEM images.

Note: To examine any morphological changes that may accompany the aging of the SWCNT-TiO_2 dispersion, SEM images were obtained at various time intervals. Taken after its initial preparation, SEM images in Figure S6 show the polydispersity of the SWCNT-TiO_2 material. Specifically, TiO_2 spheres and cylindrical rods are interconnected by bundles of SWCNTs, thereby creating an interwoven, bridging network of electronic transport highways. Over the course of 4 weeks, the morphology of SWCNT-TiO_2 changed from rod-like to more predominantly spherical with a concomitant increase in surface roughness and hence surface area (Figure S7). SEM images were also collected for **SWCNT-TiO_2-Re**, which exhibits morphology changes similar to those observed for SWCNT-TiO_2 (Figure S8). The sizes of the constituent TiO_2 particles were estimated at different timepoints in dispersion, which shows that the average particle diameter increases over the time, likely due to Ostwald ripening (Figure S9). In parallel with a growth in TiO_2 particle size, SWCNTs bundling is also observed, with more exposed segments after 4 weeks. Taken together, these morphological changes suggest a general increase in the analyte-accessible surface area of **SWCNT-TiO_2-Re** and may account for its increased sensing performance after a 4-week aging period.

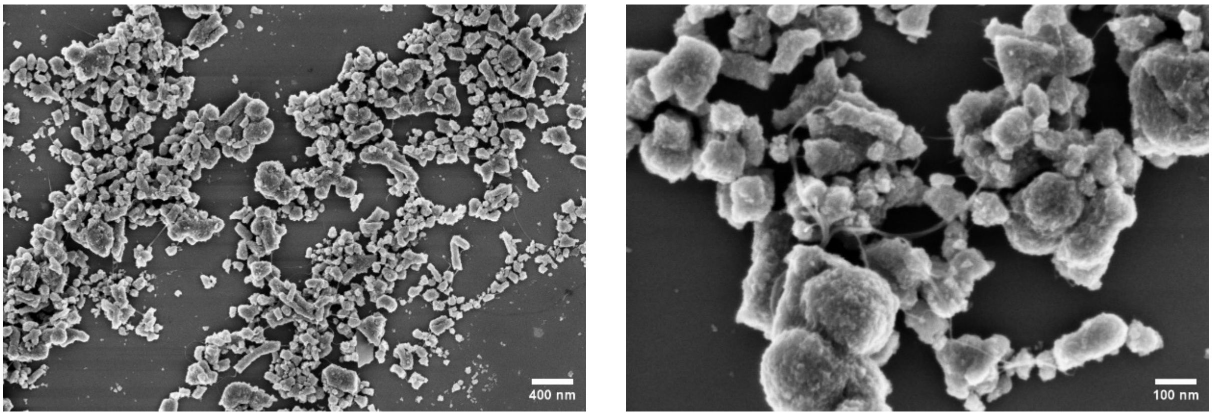

Figure S6. SEM images of freshly dispersed SWCNT-TiO_2 spray-coated onto a silicon wafer.

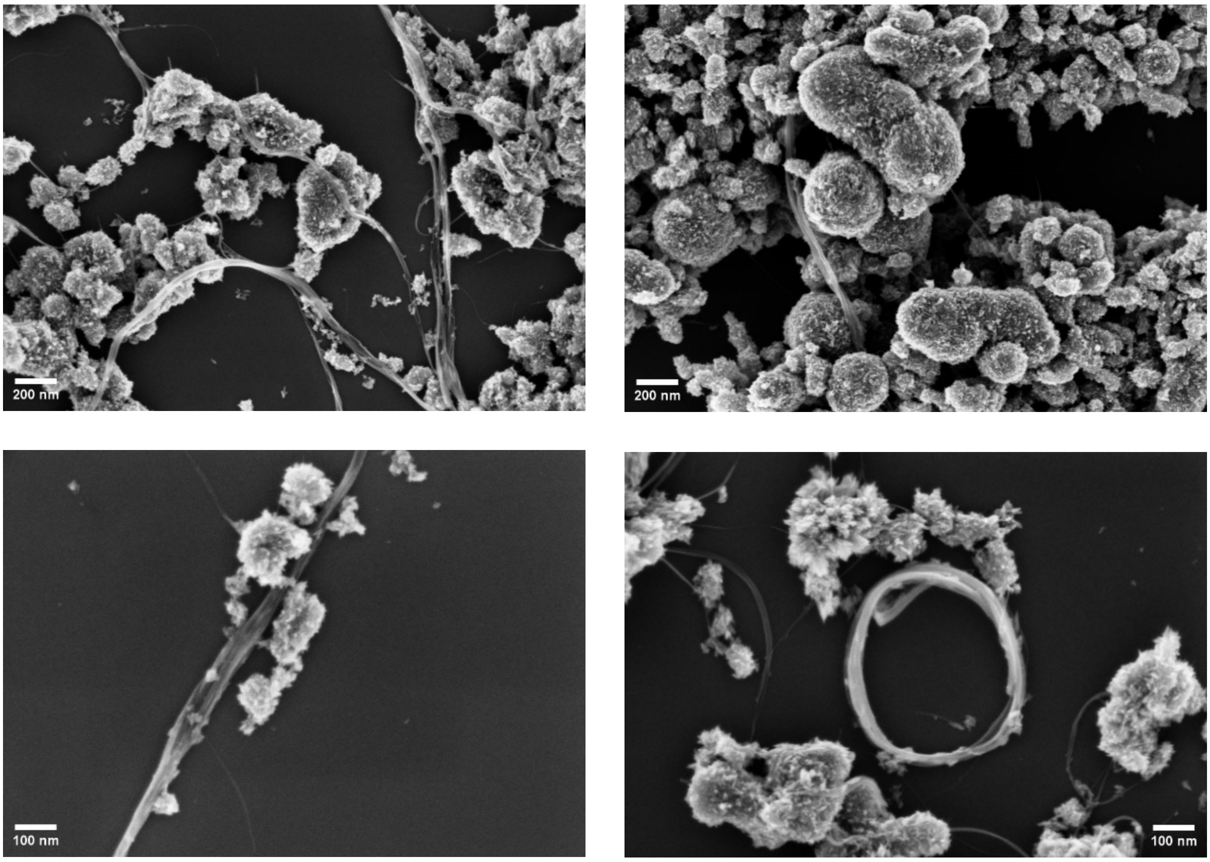

Figure S7. SEM images of SWCNT-TiO_2 spray-coated onto a silicon wafer after storage for 4 weeks in a water dispersion.

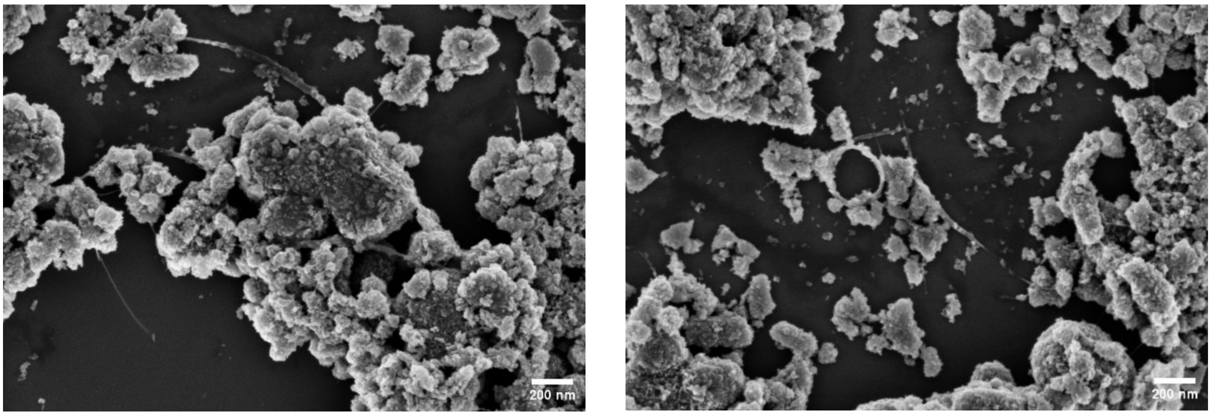

Figure S8. SEM images of **SWCNT-TiO_2-Re** spray-coated on a silicon wafer after storage for 4 weeks in a water dispersion.

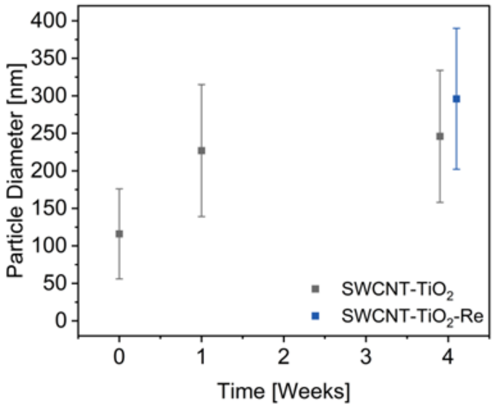

Figure S9. Comparison of TiO_2 diameter in SWCNT-TiO_2 hybrid after different times in a water dispersion. At the timepoint of 4 weeks in dispersion, SWCNT-TiO_2 (grey) as well as **SWCNT-TiO_2-Re** composite (blue) are shown.

Powder X-ray Diffraction

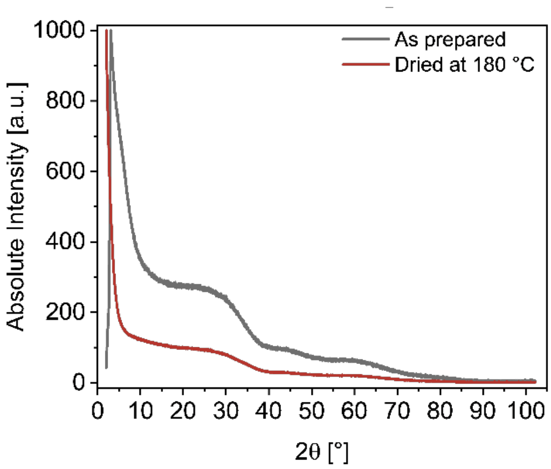

Figure S10. Powder X-ray diffractogram of SWCNT-TiO_2 after preparation and after drying at 180 °C overnight.

IR Spectroscopy

Sample Preparation

For [(^{P}bpy)(CO)$_3$ReBr] and SWCNT-TiO_2, IR spectra were obtained by grinding approximately 1 mg of the solid materials and pressing them into pellets with excess KBr (approximately 100 mg). To obtain IR spectra of **SWCNT-TiO$_2$-Re**, a solution of [(^{P}bpy)(CO)$_3$ReBr] in DMSO (0.3 mL, 2.0 mM) was added dropwise to an aqueous dispersion of SWCNT-TiO_2 (2 mL, 0.75 mg/mL) under vigorous stirring. After 1 h, the dispersion was filtered over a Nylon filter (0.2 µm), washed with DMSO (2 × 1.5 mL) and water (2 drops), and the filtrate was dried under vacuum for 4 h. The dried product was mixed with KBr (ca. 100 mg) and ground into a fine powder and pressed into a pellet.

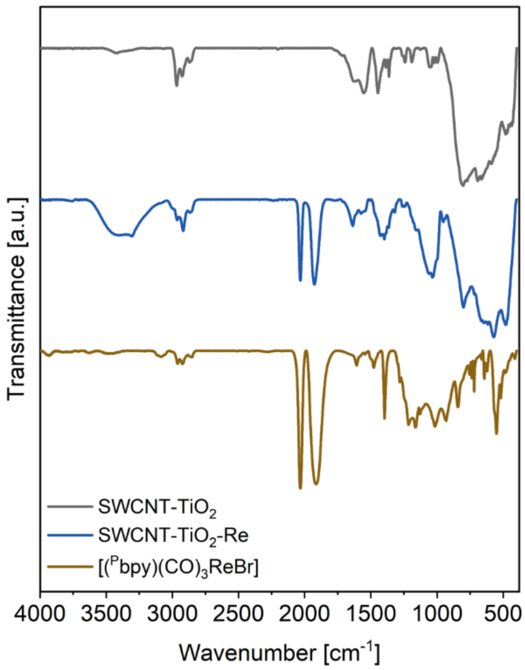

Figure S11. Baseline-corrected IR spectra of **SWCNT-TiO$_2$-Re** (blue) together with SWCNT-TiO_2 (grey) and [(^{P}bpy)(CO)$_3$ReBr] (gold). The bands just below 3000 cm^{-1} are background signals which were also observed when measuring a plain KBr pellet.

UV-vis DRS

Sample Preparation

A concentrated aqueous dispersion of SWCNT-TiO_2 (1.0 mL, 4 mg/mL) was drop-cast on an Epredia frosted microscope slide at 150 °C. The slide was cooled down to room temperature, and the sensitizer was introduced by applying 6 drops of a solution of [(^{P}bpy)$(CO)_3$ReBr] in DMSO (2.0 mM). The glass slide with the solution on top was left at room temperature overnight, before it was washed with DMSO (6 drops) as well as water (6 drops) and dried under high vacuum for 3 h.

Band Gap Determination for SWCNT-TiO_2

SWCNT-TiO_2 exhibits a sharp increase in reflectance at approximately 375 nm. The presence of [(^{P}bpy)$(CO)_3$ReBr] causes the appearance of an additional shoulder at around 400 nm. The obtained spectra of SWCNT-TiO_2 were analyzed by the DASF and Tauc methods. An overview of the employed methods is given in Figure S12. Since the classical Tauc method only yields reasonable estimates of the band gap for bare semiconducting substrates that show a negligible absorption below the band gap region but not for composite materials such as SWCNT-TiO_2, a modified Tauc method was applied to determine the band gap. Hence, the intersection of the linear fit of the Tauc plot with a "baseline" was used to estimate the band gap.[3,4] With the advantage of avoiding the necessity to know the absorption coefficient *α* and the type of band gap transition, the DASF method was used as a complementary approach. According to the DASF method, the band gap yields a discontinuity in a plot of $\frac{d}{d(1/\lambda)}\ln\left(\frac{A(\lambda)}{\lambda}\right)$ vs $(1/\lambda)$ at the band gap energy.[5,6,7]

Data Treatment Workflow

The raw UV-vis DRS data was corrected for a sharp increase in reflectance at 570 nm. Subsequently, the data was smoothened using the moving average filter with a window length of 15 points, before converting to the absorbance by the Kubelka–Munk function shown below.

$$F(R) = \frac{(1-R)^2}{2R}$$

In this equation, *R* is the experimentally measured reflectance. For the DASF method, the first derivative of the logarithm of the ratio of the Kubelka–Munk function and the wavelength, ln(F(R)/λ), was taken. In order to find the maximum, the latter was smoothened again by the same filter, using a window length of 10 points. Plotted are the non-smoothened derivatives. The Tauc method (see below) was applied to the Kubelka-Munk function, assuming both, an allowed direct (*γ* = *1/2*) and an allowed indirect (*γ* = *2*) band gap. Plotting $(F(R)\cdot h\nu)^{1/\gamma}$ as a function of photon energy $h\nu$ yields the Tauc plot, with a linear increase in absorption above the band gap energy E_g, where *B* is a constant. For the determination of the band gap, the intersection of a linear fit in this region with a baseline was used.[4,5]

$$(F(R)\cdot h\nu)^{1/\gamma} = B(h\nu - E_g)$$

While anatase is typically reported as indirect semiconductor, a direct band gap is commonly accepted for rutile.[8,9] However, since the TiO_2 phase in SWCNT-TiO_2 was found to be amorphous , the type of transition is not known. The obtained Tauc plots did not clearly indicate

whether SWCNT-TiO_2 features a direct or an indirect band gap and the analysis was hence performed assuming both a direct and indirect band gap, yielding 3.53 eV and 3.33 eV, respectively. The DASF method yielded a band gap of 3.52 eV.

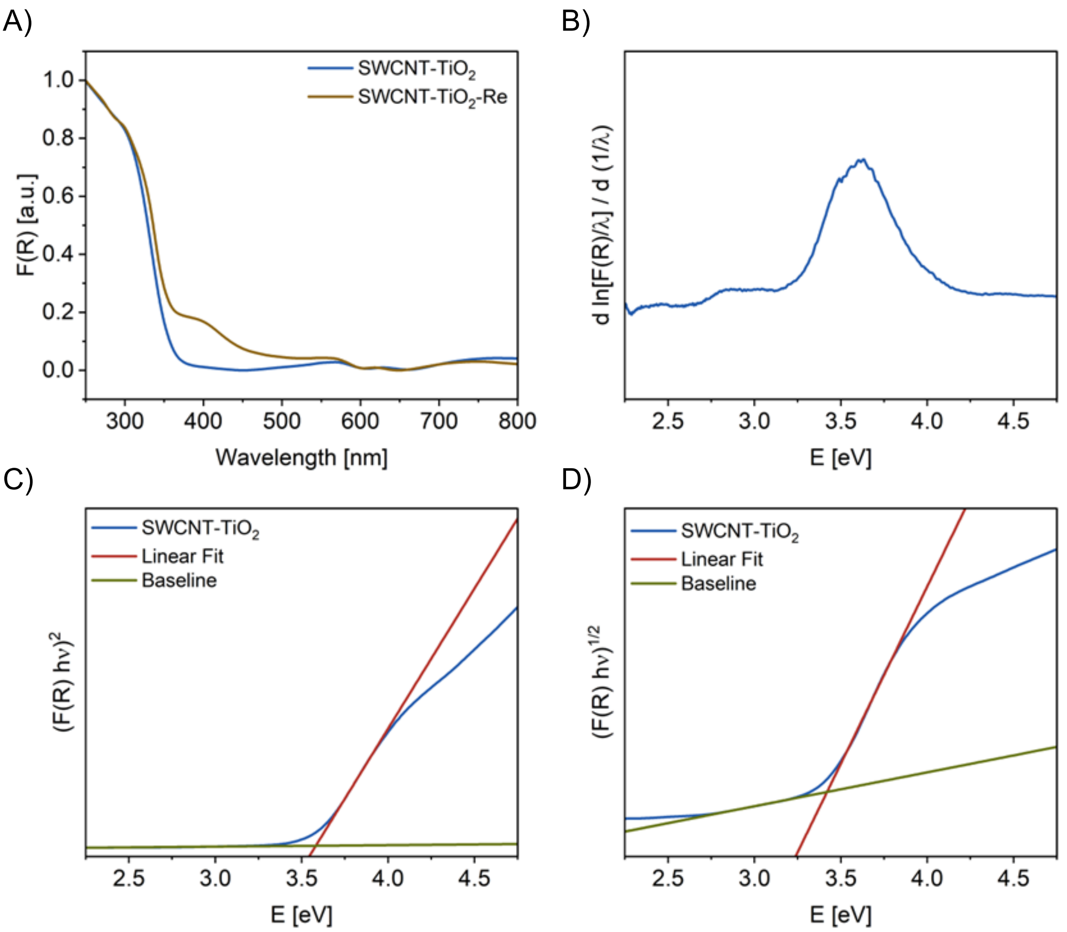

Figure S12. A) UV-vis DRS spectra of SWCNT-TiO_2 (blue) and **SWCNT-TiO_2-Re** (bronze). B) DASF plot of SWCNT-TiO_2 (blue). The peak at 3.52 eV was used to determine the band gap. C) Tauc plot for SWCNT-TiO_2 (blue) assuming a direct band gap ($\gamma = 1/2$). The band gap was obtained from the intersection of the linear fit (red) with a baseline (green), yielding 3.53 eV. D) Tauc plot for SWCNT-TiO_2 (blue) assuming an indirect band gap ($\gamma = 2$). The band gap was obtained from the intersection of the linear fit (red) with a baseline (green), yielding 3.33 eV.

Table S1. Summary of band gap energies (E_g) of SWCNT-TiO_2 determined from different methods.

	**DASF**	**Tauc ($\gamma = 1/2$)**	**Tauc ($\gamma = 2$)**
E_g [eV]	3.52	3.53	3.33

XPS

Sample Preparation

A concentrated aqueous dispersion of SWCNT-TiO_2 (1.0 mL, 4 mg/mL) was drop-cast on an ITO coated glass slide at 150 °C. The slide was cooled down to room temperature, and the sensitizer was introduced by applying 6 drops of a solution of [(^{P}bpy)(CO)$_3$ReBr] in DMSO (2.0 mM). The glass slide with the solution on top was left at room temperature overnight, before it was washed with DMSO (6 drops) as well as water (6 drops) and dried under high vacuum for 3 h.

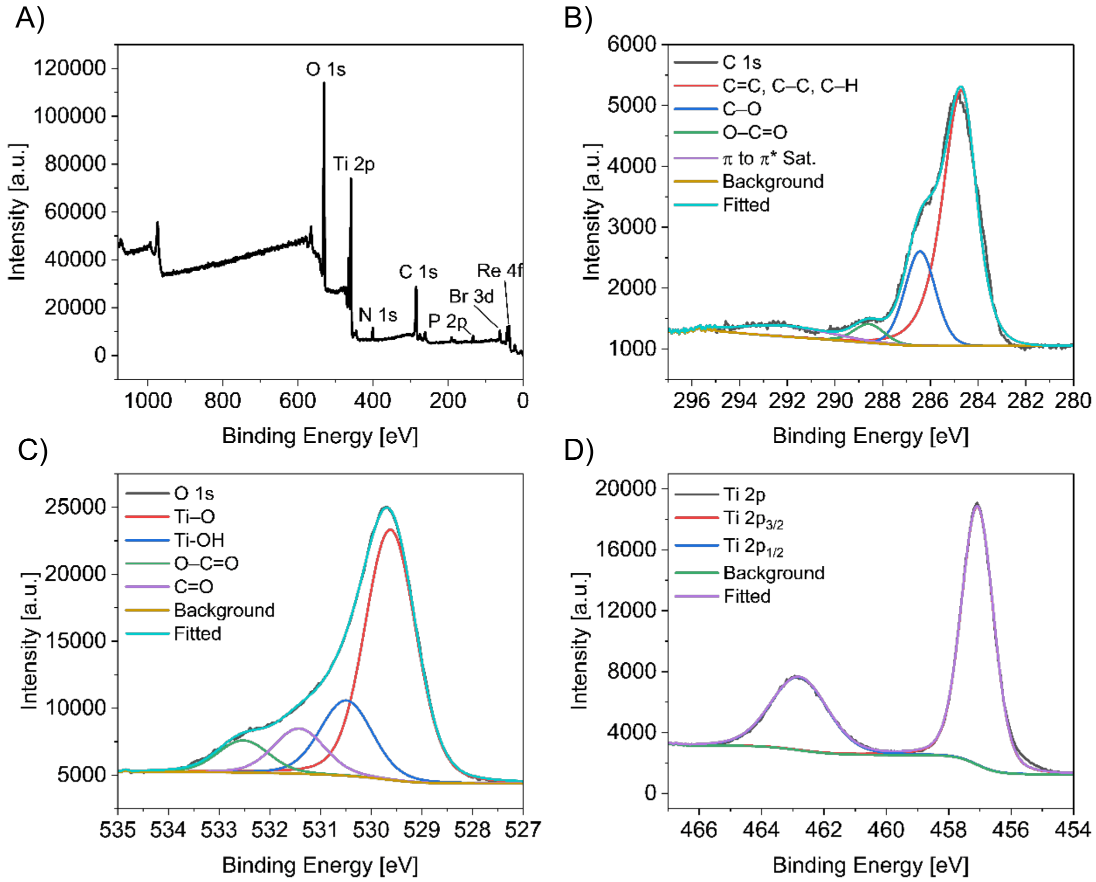

Figure S13. A) Survey XPS spectrum of **SWCNT-TiO_2-Re** film on ITO glass substrate. B–D) High-resolution XPS spectra of **SWCNT-TiO_2-Re** film on ITO glass substrate.

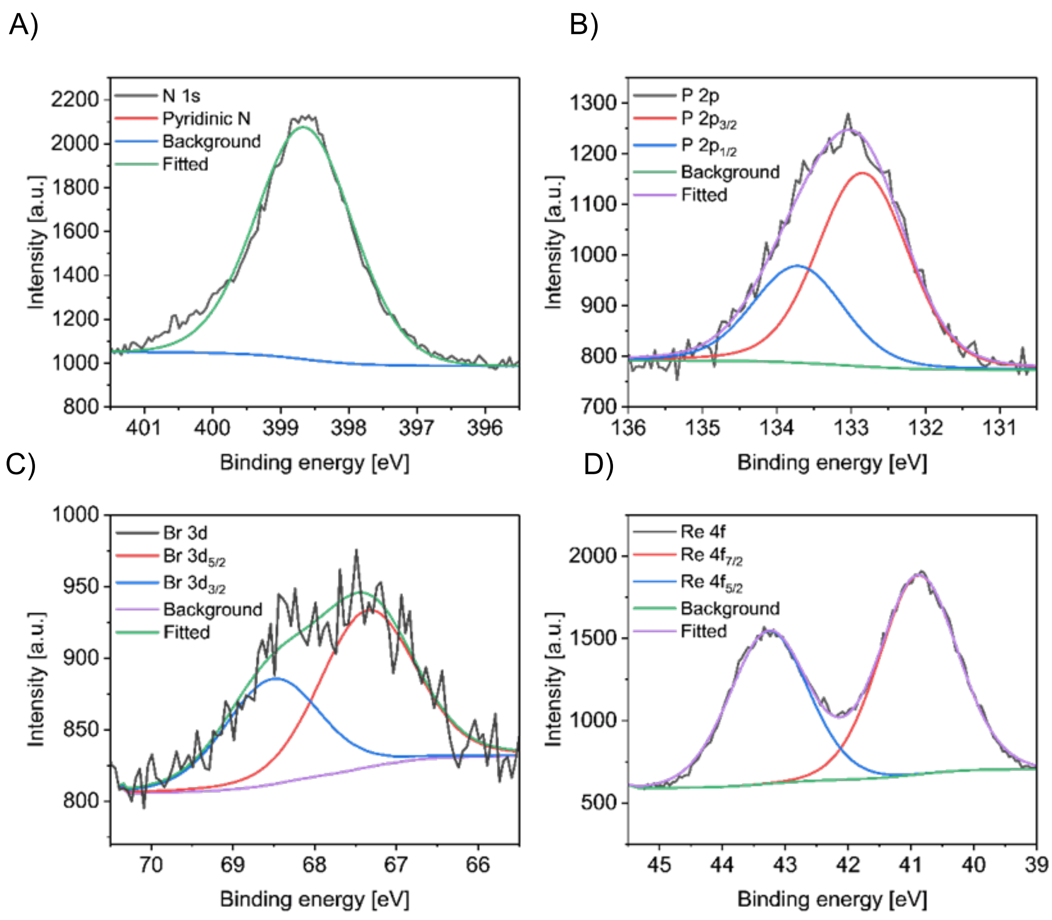

Figure S14. A–D) High-resolution XPS spectra of **SWCNT-TiO$_2$-Re** film on ITO glass substrate.

Raman Spectroscopy

Sample Preparation

Raman spectra of **SWCNT-TiO_2-Re** were collected directly on the sensing electrode prepared according to the standard procedure described above ("Preparation of SWCNT-TiO_2-Re").

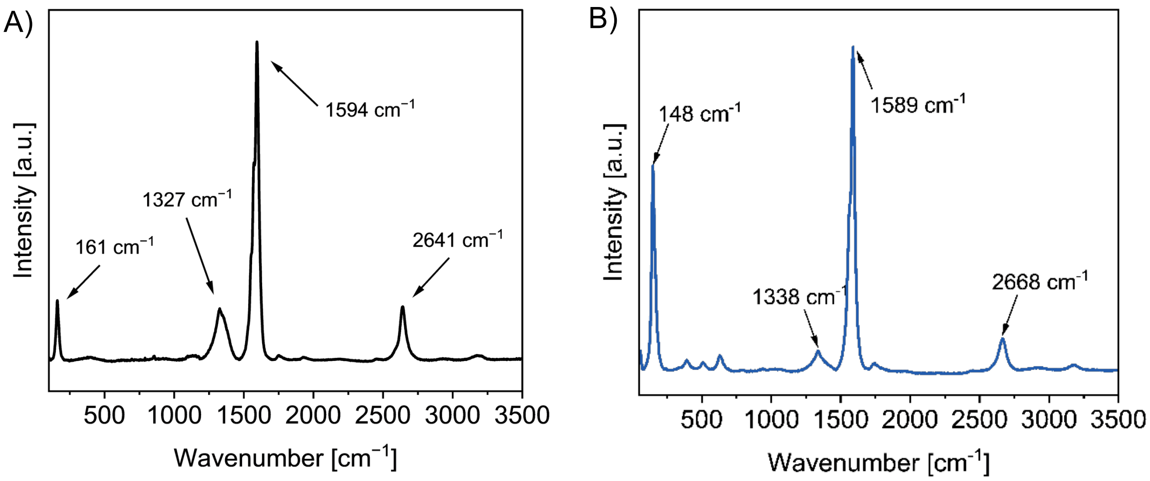

Figure S15. A) Baseline-corrected Raman spectrum of as-purchased SWCNT. B) Baseline-corrected Raman spectrum of **SWCNT-TiO_2-Re** deposited on an interdigitated electrode. The spectra were collected using a laser excitation wavelength of λ_{ex} = 532 nm.

IV. O_2 Detection Measurements

Chemiresistive sensing measurements were carried out using a custom-made sensing platform shown in Figures S16-S17. Interdigitated electrodes bearing the **SWCNT-TiO_2-Re** film were inserted into a custom-made sensing board containing a 2 × 17 pin edge connector (TE Connectivity: CONN SEC II 17 POS 100C/L, Figure S16A). The edge connector containing the electrodes was then sealed inside of a custom-made PTFE chamber featuring a sealed glass window and openings for gas inlet and exhaust (Figure S16B). The sensing board was connected to an Agilent Keysight DAQ970A data collection unit equipped with a DAQM901A 20 channel multiplexer (2/4 wire) module using a ribbon cable. Using Agilent BenchLink Data Logger 3 software, resistance values were recorded during the sensing measurements. The scan rate was set to 1 scan per second.

Gases were introduced to the PTFE chamber by connection to a gas flow-controlling platform via stainless steel tubing (Swagelok, Figure S17). Gas flow and concentration were adjusted using mass-flow controllers (MFCs, Alicat Scientific). Three MFCs (MC-5SLPM-D/5V) were used to deliver the carrier gas (N_2) at controlled relative humidity with a total flow rate of 1.00 L/min. Relative humidity was adjusted by controlling the relative ratios of dry and water-saturated carrier gas streams. A humidity sensor (Sensirion: SEK-SHT45-AD1B-SENSORS) was used to calibrate relative humidity. The analyte gas concentration was controlled using a finely adjustable Alicat Scientific MFC (MC-100SCCM-D/5V).

The electrodes were irradiated by an LED through the sensing chamber glass window from a distance of 2.0 cm (Figure S16C). The LED was purchased from LCFOCUS (LC-10RGB-C30). The LED current was set to 0.08 A for all measurements using a VOLTCRAFT LPS 1305 power supply, which corresponds to an irradiance of approximately 200 mW/mm^2.

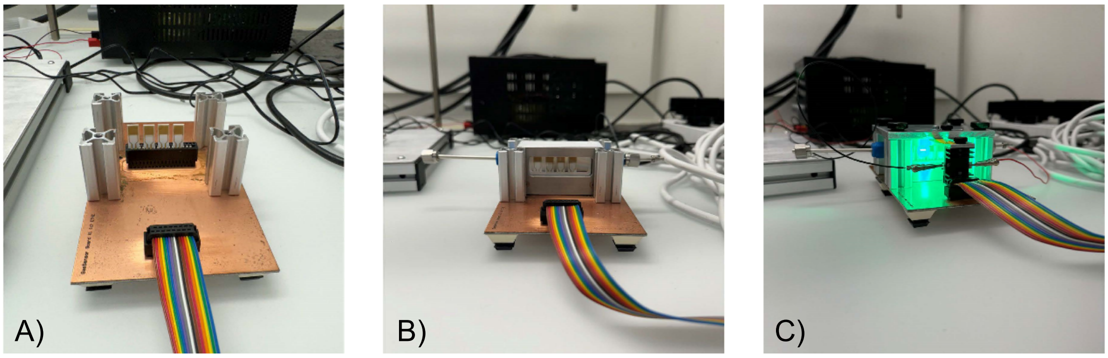

Figure S16. A) Four chemiresistor devices inserted into the edge-connector mounted on the sensing board with a ribbon cable connection. B) Gas-tight PTFE chamber with window fitted onto the platform. C) Green LED irradiating the chemiresistor devices.

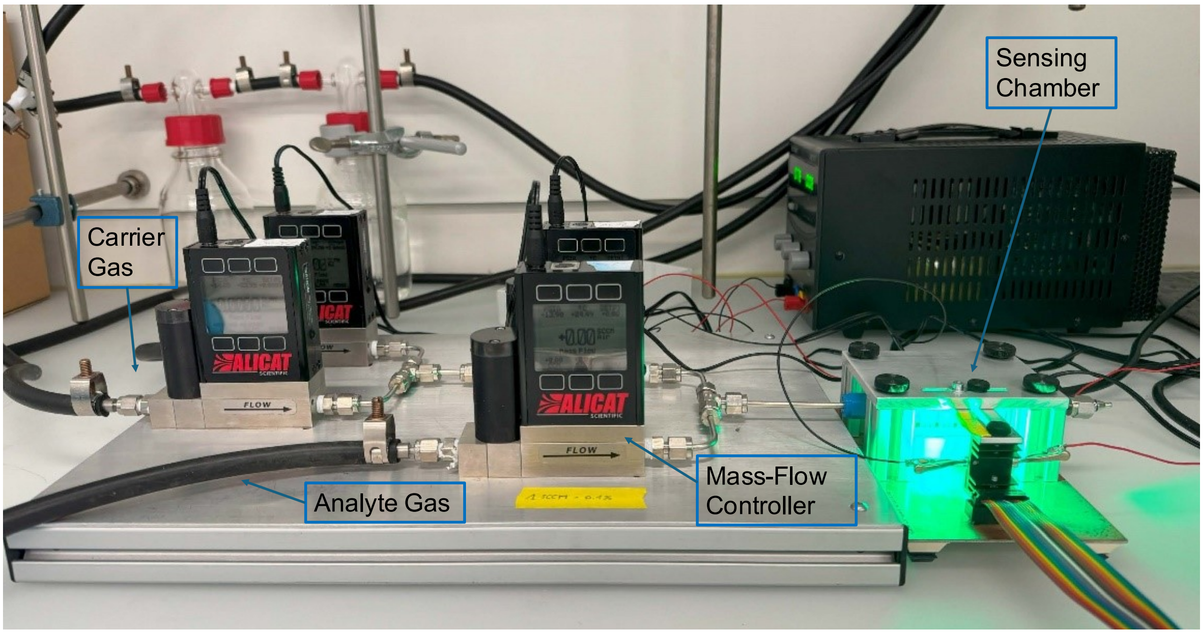

Figure S17. Assembled chemiresistive O_2 sensing setup.

The change in device resistance resulting from O_2 exposure was converted to the normalized change in resistance [$-\Delta R/R_0 = -(R-R_0)/R_0 \times 100\%$; R_0 = initial resistance]. All sensing data is reported as an average of at least 3 separate devices and is depicted with shaded areas (for sensing traces) and error bars (for bar graphs) to show standard deviations.

Prior to the start of sensing experiments, the devices were equilibrated under 1.00 SLM carrier gas flow and light irradiation for 15 minutes to ensure a stable baseline. A drift correction was applied in cases where the absolute response was of interest using linear regression fitting of the baseline (Figure S18).

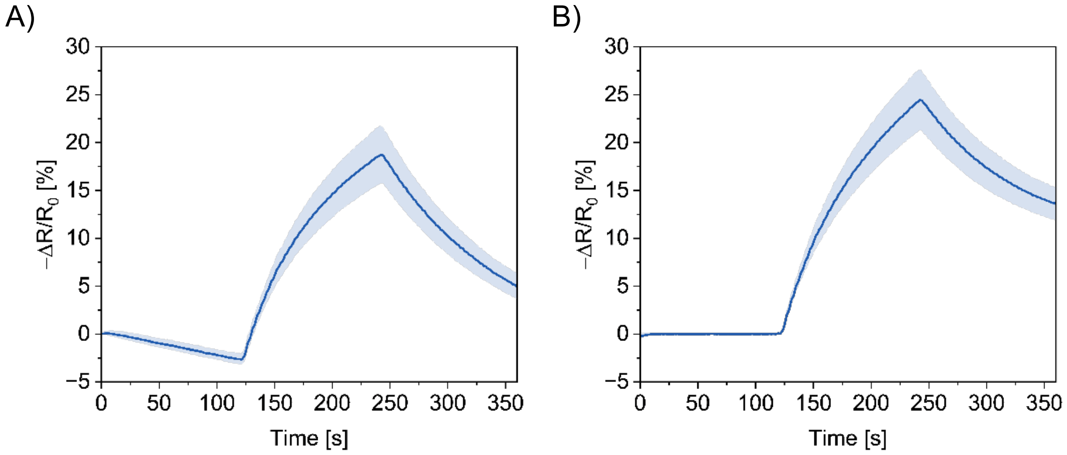

Figure S18. Normalized change of resistance of a typical sensing experiment A) before and B) after baseline drift correction.

V. Additional Sensing Data

Sensor Response Time Estimate

The response time of the sensor was estimated by analyzing the point of greatest resistance change after exposure to 1000 ppm O_2. For this purpose, the first derivative of the normalized sensing data was plotted, with response time defined as the point at which the rate of change in normalized resistance reaches its maximum value.

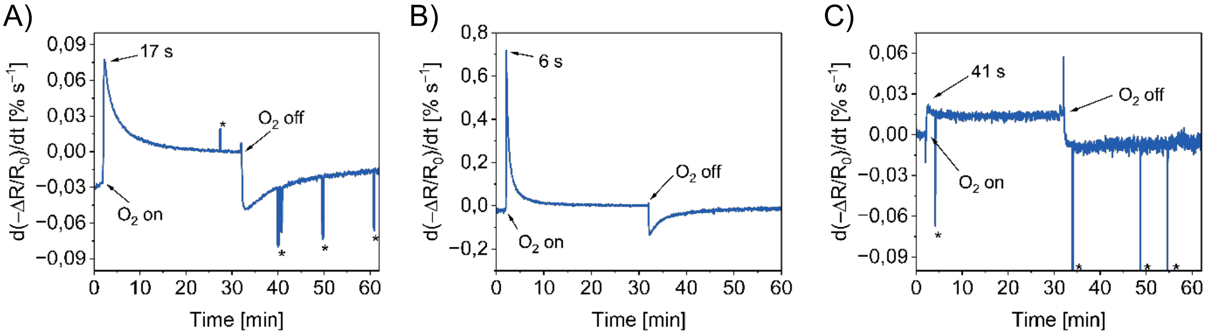

Figure S19. First derivative of the normalized resistance against time upon exposure to 1000 ppm O_2 for A) SWCNT-TiO_2 under green light illumination, B) **SWCNT-TiO_2-Re** under green light illumination, and C) **SWCNT-TiO_2-Re** in the dark. Electronic signal artefacts are labelled with an asterisk (*).

Sensor Recovery Time Estimate

The recovery time of the sensor was estimated by analyzing the time necessary for the baseline drift to reach its initial value after exposure to 1000 ppm O_2 for 2 min (Figure S20A). For this purpose, the first derivative of the normalized sensing data was plotted, with baseline recovery defined as the point at which the rate of change in normalized resistance returns to the initial value (Figure S20B).

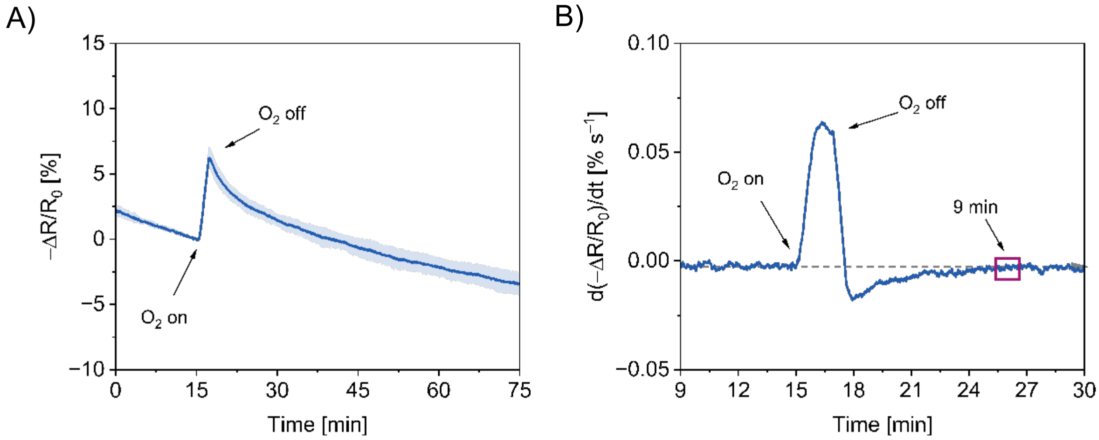

Figure S20. A) Normalized resistance trace of a 2 min exposure of **SWCNT-TiO_2-Re** to 1000 ppm O_2. B) First derivative of the resistance against time indicating baseline recovery after 9 min.

Sensor Optimization

SWCNT-TiO2 Deposition Method

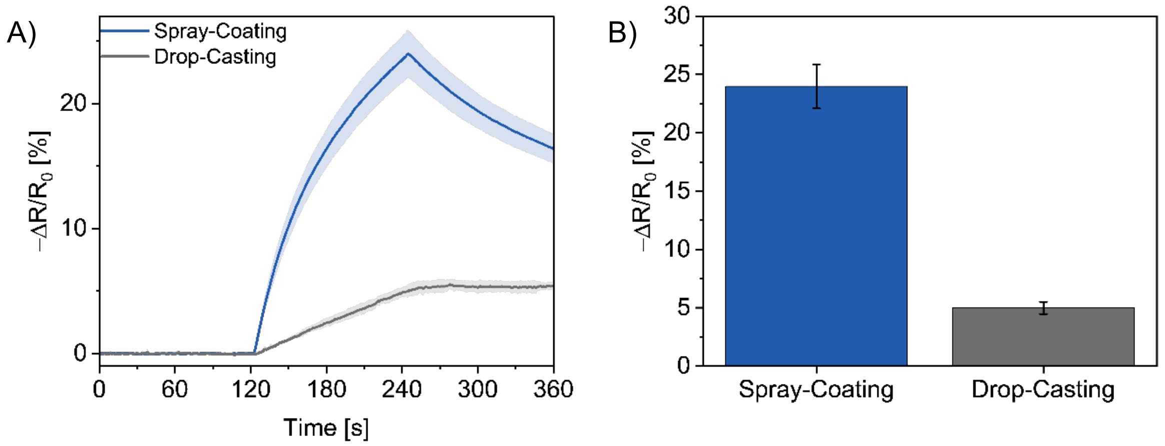

Figure S21. A) Normalized and drift-corrected resistance traces for 2 min exposure of **SWCNT-TiO2-Re** to 1000 ppm O_2 prepared by spray-coating and drop-casting. B) Data represented as bar graphs. Shaded areas and error bars represent standard deviations (*N* = 4 chemiresistors); all data were collected under green light irradiation at room temperature using dry nitrogen carrier gas (R.H. = 0%).

Initial Device Resistance

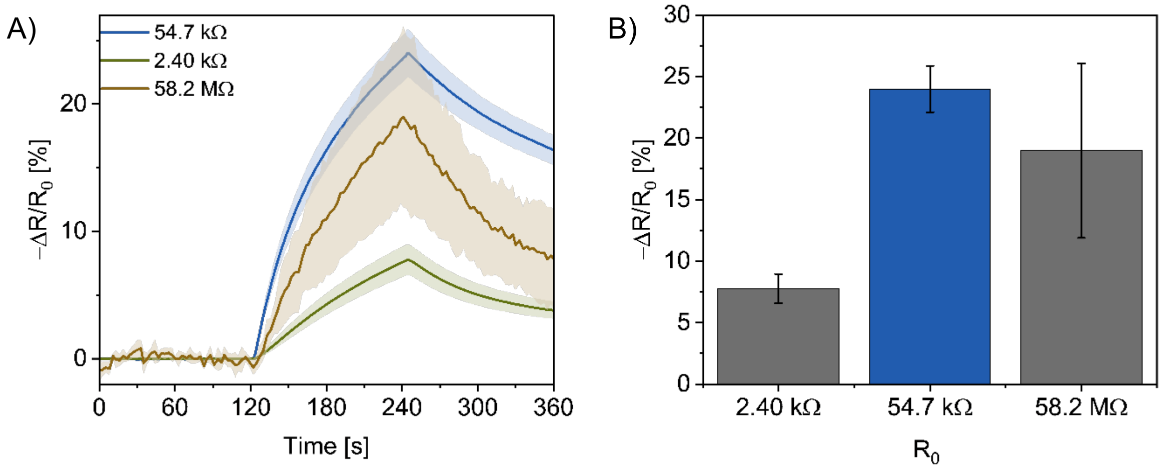

Figure S22. A) Normalized and drift-corrected resistance traces for 2 min exposure of **SWCNT-TiO2-Re** to 1000 ppm O_2 featuring different initial device resistances (R_0). R_0 refers to device resistance following equilibration under 1.00 SLM carrier gas flow and green light irradiation for 15 minutes. B) Data represented as bar graphs. Shaded areas and error bars represent standard deviations (*N* = 4 chemiresistors); all data were collected under green light irradiation at room temperature using dry nitrogen carrier gas (R.H. = 0%).

TiO_2 Phase

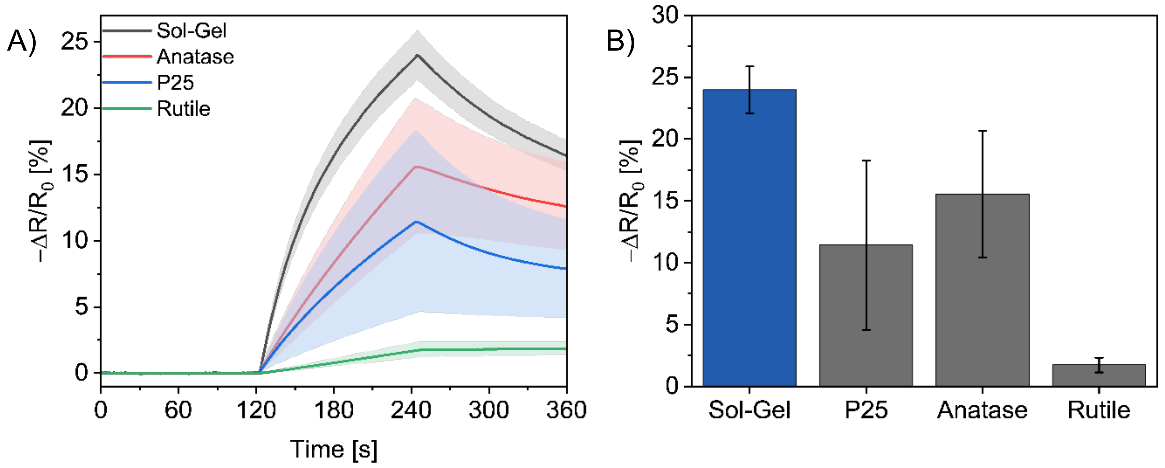

Figure S23. A) Normalized and drift-corrected resistance traces for 2 min exposure of **SWCNT-TiO_2-Re** to 1000 ppm O_2 featuring different phases of TiO_2. "Sol-Gel" refers to SWCNT-TiO_2 prepared by the sol-gel method described above ("Preparation of SWCNT-TiO_2-Re"), while other TiO_2 phases were mechanically mixed with SWCNTs. B) Data represented as bar graphs. Shaded areas and error bars represent standard deviations (N = 4 chemiresistors); all data were collected under green light irradiation at room temperature using dry nitrogen carrier gas (R.H. = 0%).

Concentration of Re-Sensitizer in Soaking Solution

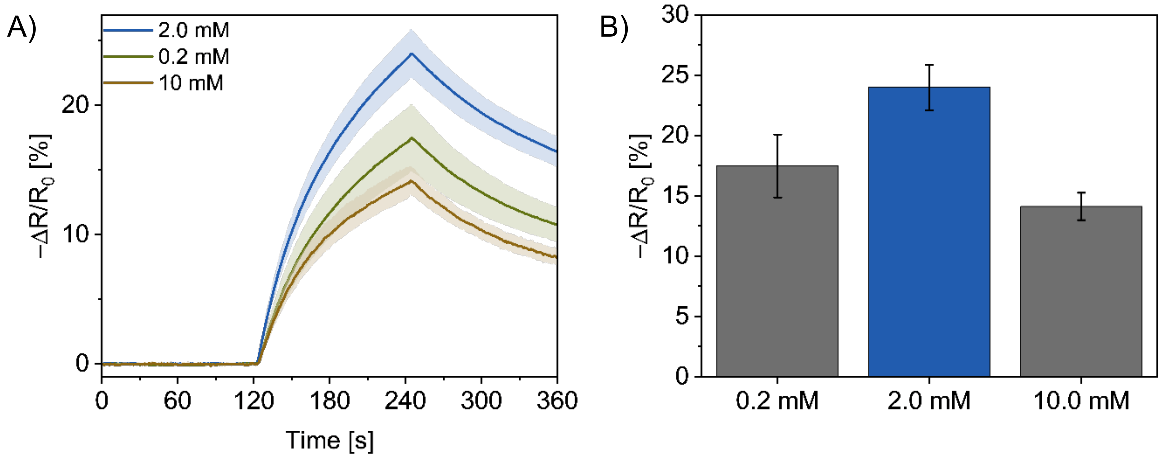

Figure S24. A) Normalized and drift-corrected resistance traces for 2 min exposure of **SWCNT-TiO_2-Re** to 1000 ppm O_2 prepared using different concentrations of sensitizer in the soaking solution. B) Data represented as bar graphs. Shaded areas and error bars represent standard deviations (N = 4 chemiresistors); all data were collected under green light irradiation at room temperature using dry nitrogen carrier gas (R.H. = 0%).

Control Experiments

Systematic Omission of Chemiresistor Components

The O_2 detection performance of the active sensing material **SWCNT-TiO$_2$-Re** under green light illumination was compared to modified versions that omitted one or more components (Figure S25). Application of TiO_2 (without SWCNT), prepared by the sol-gel method described above ("Preparation of SWCNT-TiO$_2$-Re") leads to no measurable conductance, and the same holds true for TiO_2-Re. Therefore, no chemiresistive sensing experiment could be performed. In the case of SWCNT-TiO$_2$, a response to 1000 ppm of O_2 is observed. However, this response does not show any reversibility and is thus dosimetric. SWCNT-Re refers to electrodes bearing SWCNTs that were soaked in a DMSO solution of [(^{P}bpy)(CO)$_3$ReBr] overnight, then washed, and dried according to the method described above ("Preparation of SWCNT-TiO$_2$-Re").

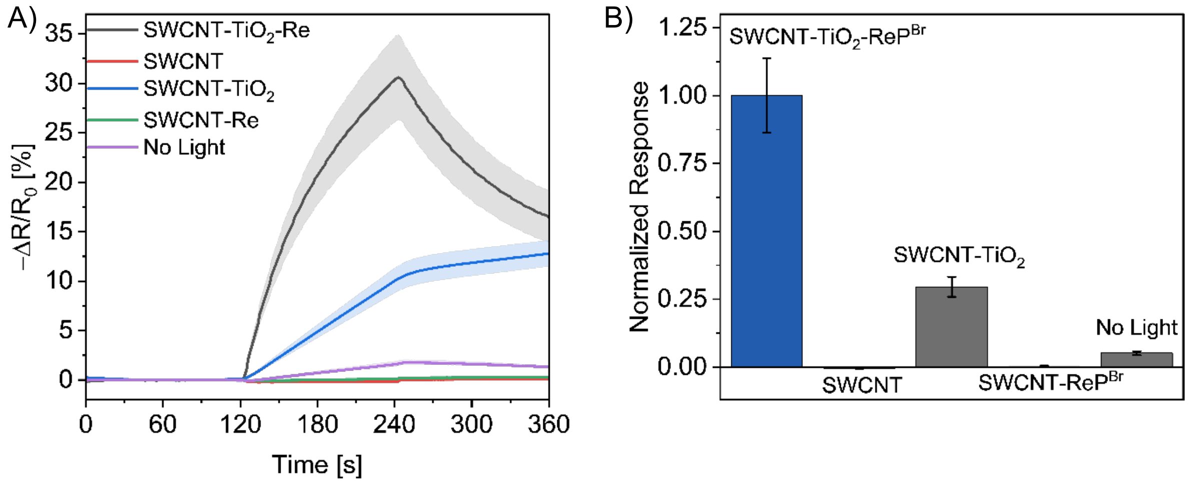

Figure S25. A) Normalized and drift-corrected resistance traces for 2 min exposure of **SWCNT-TiO$_2$-Re** to 1000 ppm O_2 compared to the control devices. B) Data represented as bar graphs. Shaded areas and error bars represent standard deviations ($N = 4$ chemiresistors); all data were collected under green light irradiation at room temperature using dry nitrogen carrier gas (R.H. = 0%).

Alternative Sensitizers

Introduction of the sensitizers N3 and N719 into the sensing composite was accomplished in analogy to **SWCNT-TiO$_2$-Re** described in Section "Preparation of SWCNT-TiO$_2$-Re", by exposing electrodes bearing SWCNT-TiO$_2$ films to DMSO solutions (2.0 mM) of N3 and N719, respectively. Identical electrode washing and drying procedures were carried out (Figure S26).

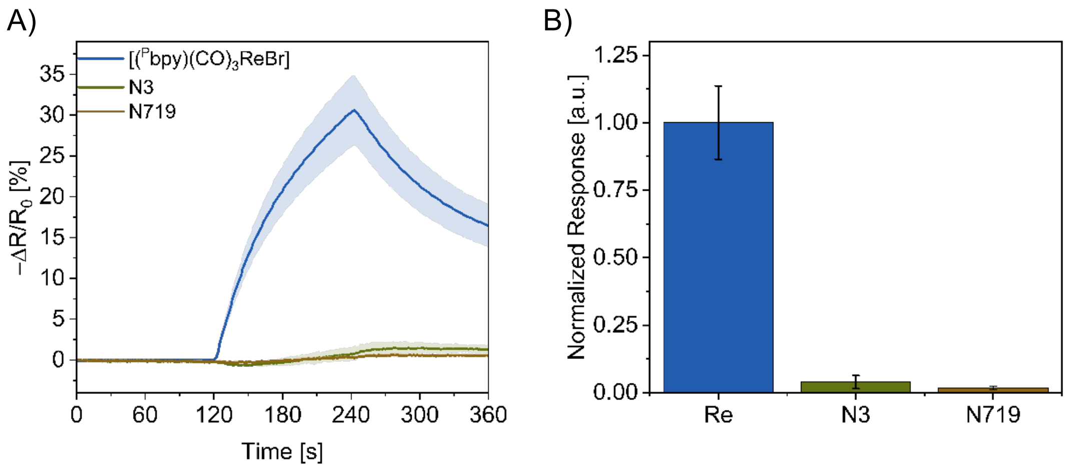

Figure S26. A) Normalized and drift-corrected resistance traces for 2 min exposure of **SWCNT-TiO$_2$-Re** to 1000 ppm O$_2$ compared to devices containing N3 and N719 as sensitizers. B) Data represented as bar graph. Shaded areas and error bars represent standard deviations (N = 4 chemiresistors); all data were collected under green light irradiation at room temperature using dry nitrogen carrier gas (R.H. = 0%).

Absolute Device Resistance Values

Typical responses of **SWCNT-TiO$_2$-Re** to green light illumination (Figure S27A) and to 1000 ppm O$_2$ under dry (Figure S27B) and humid conditions (40% R.H., Figure S27C) are presented in units of absolute device resistance.

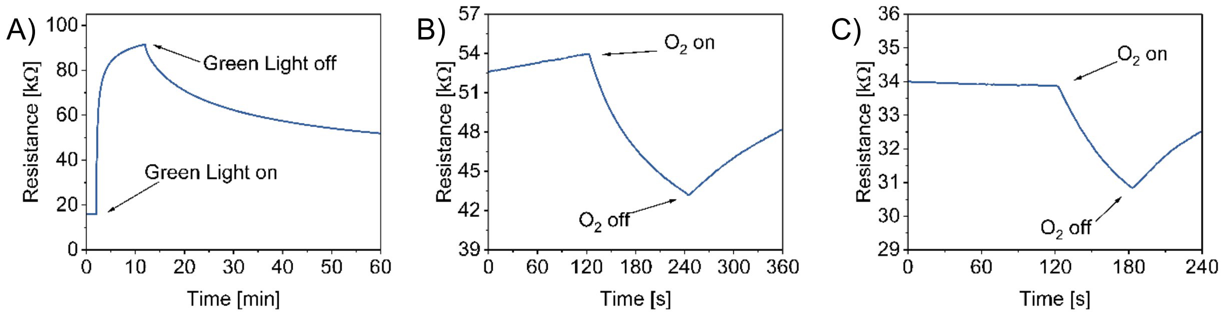

Figure S27. Absolute device resistance traces showing typical responses of **SWCNT-TiO$_2$-Re** to A) green light illumination for 10 min, B) 1000 ppm O$_2$ exposure under dry conditions for 2 min and C) 1000 ppm O$_2$ exposure under humid conditions (40% R.H.) for 2 min.

Photoresponse

Devices bearing either **SWCNT-TiO_2-Re** or SWCNT-TiO_2 were equilibrated under a flow of N_2 (1.0 SLM) in the dark for 20 minutes. Green light was then switched on for 10 min, then switched off, and the normalized change in device resistance was recorded (Figure S28).

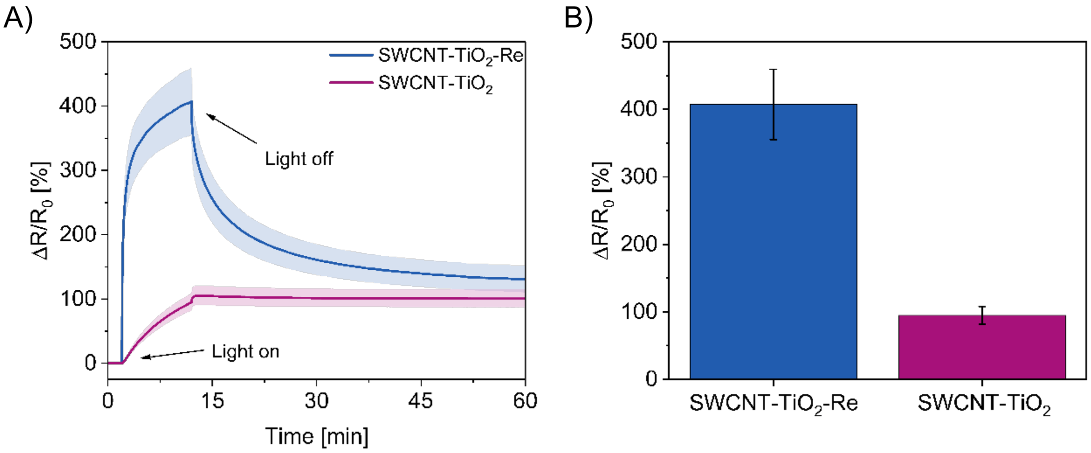

Figure S28. A) Normalized and drift-corrected resistance traces for 10 min exposure of **SWCNT-TiO_2-Re** to green light compared to SWCNT-TiO_2. B) Data represented as bar graph. Shaded areas and error bars represent standard deviations (*N* = 4 chemiresistors); all data were collected at room temperature using dry nitrogen carrier gas (R.H. = 0%).

Light Irradiance Variation

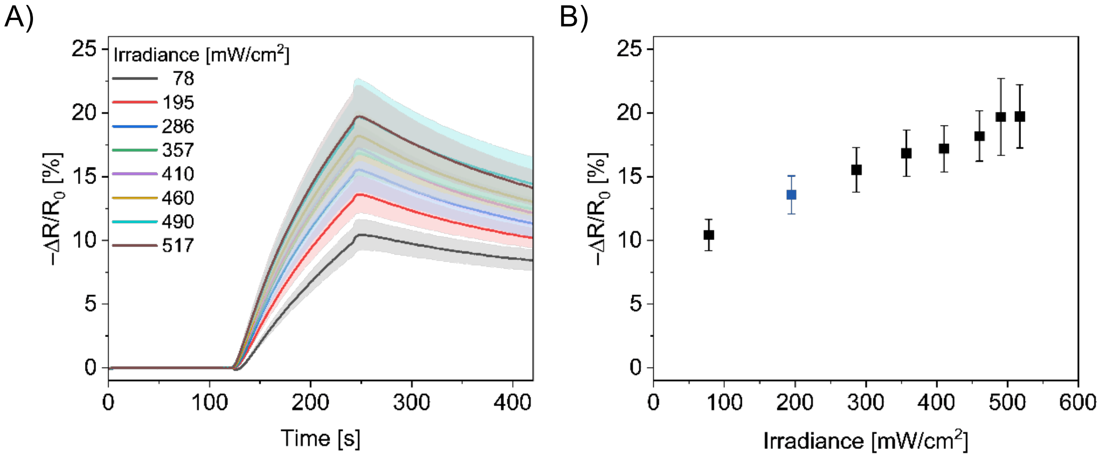

Figure S29. A) Normalized and drift-corrected resistance traces for 2 min exposures of **SWCNT-TiO_2-Re** to 1000 ppm O_2 under varying green light irradiance. B) Data represented as scatter graph, with petrol data point highlighting the irradiance used under standard sensing conditions. Shaded areas and error bars represent standard deviations (*N* = 4 chemiresistors); all data were collected at room temperature using dry nitrogen carrier gas (R.H. = 0%).

Theoretical Limit of Detection (LOD) Determination

The theoretical LOD was determined following literature procedures.[10] First, the baseline noise of the sensor was determined using the variation in the baseline signal ($\Delta R/R_0$) during 1 minute of equilibration before carrying out O_2 detection experiments. The noise was then calculated using the root-mean-square (rms) deviation of data points in this time interval. Specifically, after plotting 1 minute of the baseline data (60 data points), a fifth-order polynomial fit was executed in Microsoft Excel that gave the statistical parameters of the polynomial fit ($y = ax^5 + bx^4 + cx^3 + dx^2 + ex + f$). The parameter rms_{noise} was then calculated according to:

$$V_{\chi^2} = \sum (y_i - y)^2$$

Where y_i is an experimentally determined baseline data point and y is the corresponding value calculated using the above-determined fifth-order curve fitting equation. Then:

$$rms_{noise} = \sqrt{V_{\chi^2}/N}$$

Where N is the number of baseline data points (N = 60). According to the IUPAC definition of LOD, a true signal is when the signal-to-noise ratio is 3 or higher.[11] The LOD was thus calculated:

$$LOD\ [ppm] = 3 \times \frac{rms_{noise}}{slope}$$

Where the slope was determined from a linear regression fit of the sensor response vs O_2 concentration plot.

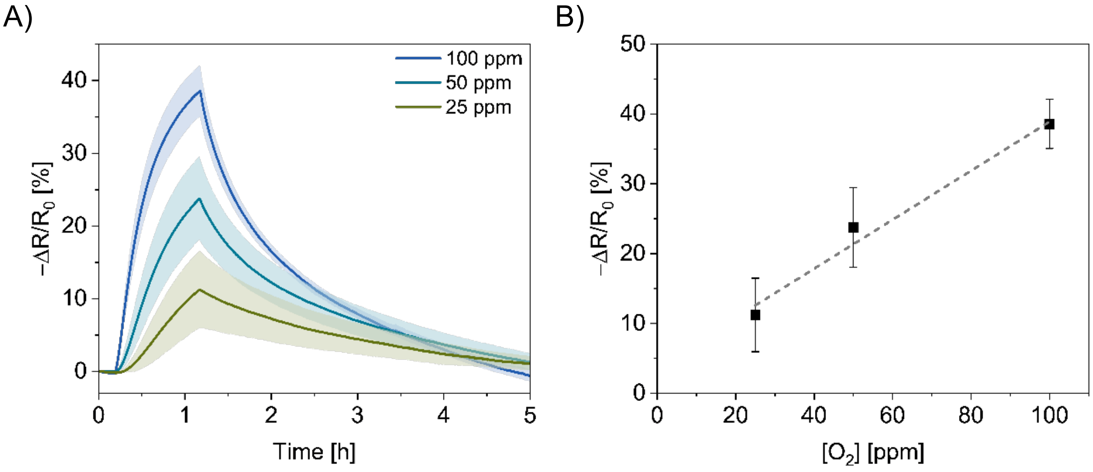

Figure S30. A) Normalized resistance traces for 60 min exposure of **SWCNT-TiO$_2$-Re** to various concentrations of O_2. B) Data represented as scatter plot and linear fit (grey) for slope determination. Shaded areas and error bars represent standard deviations (N = 4 chemiresistors); all data were collected under green light irradiation at room temperature using dry nitrogen carrier gas (R.H. = 0%).

Sensor Response to High O_2 Concentrations

Upon exposure of the sensor to higher concentrations of O_2, saturation effects are observed. The response in this regime can be approximated using a two-term exponential fit (R^2 = 0.99996):

$$y = -9.082e^{-x/552.874} - 21.773e^{-x/6414.148} + 33.144$$

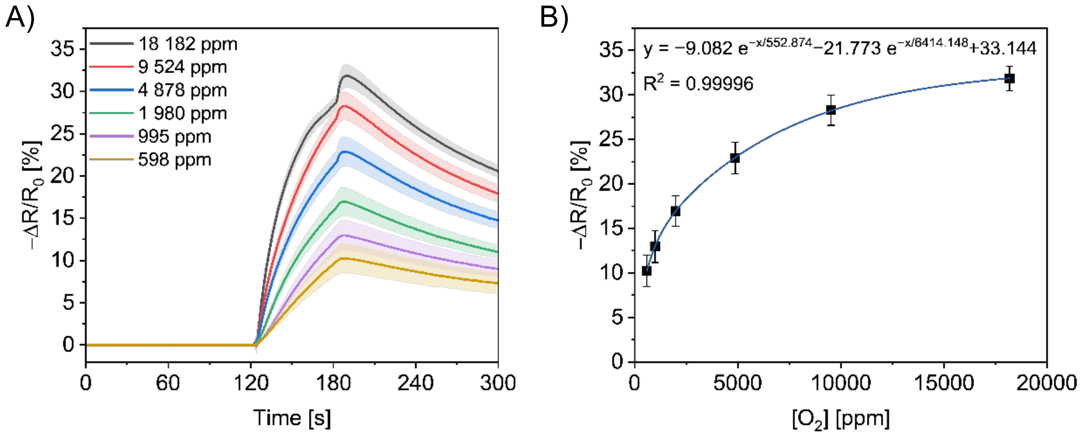

Figure S31. A) Normalized and drift-corrected resistance traces for 1 min exposure of **SWCNT-TiO2-Re** to various concentrations of O_2. B) Data represented as scatter plot. Shaded areas and error bars represent standard deviations (*N* = 4 chemiresistors); all data were collected under green light irradiation at room temperature using dry nitrogen carrier gas (R.H. = 0%).

Humidity Tolerance

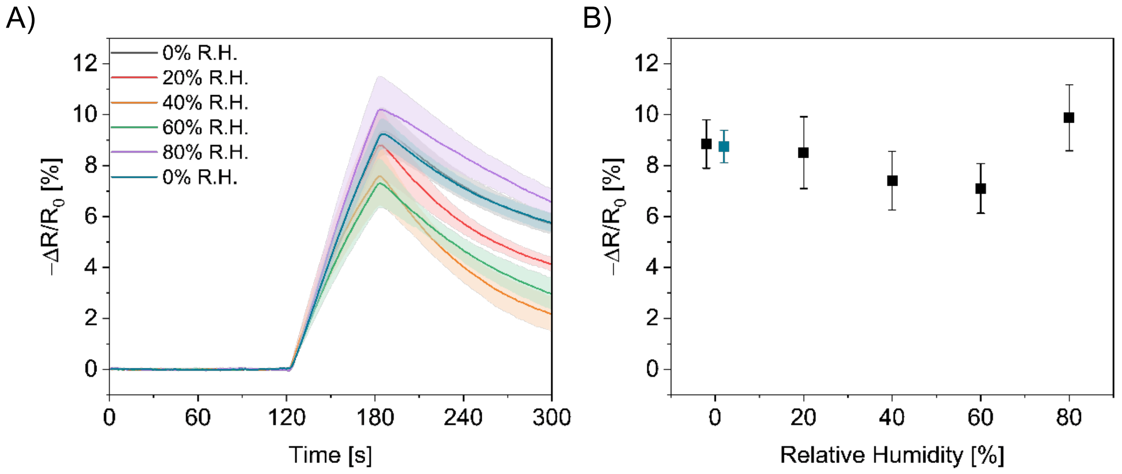

Figure S32. A) Normalized and drift-corrected resistance traces for 1 min exposure of **SWCNT-TiO2-Re** to 1000 ppm O_2 under various R.H. conditions. B) Data represented as scatter plot. The data point shown in petrol was collected immediately after the experiment at 80% RH to rule out sensor degradation upon exposure to high humidity levels. Shaded areas and error bars represent standard deviations (*N* = 4 chemiresistors); all data were collected under green light irradiation at room temperature.

Selectivity Study

Note: Before starting a sensing experiment involving a different analyte gas, the analyte channel tubing was purged for at least 15 minutes at 100 SCCM. In the case of CO, a detector was placed next to the fume hood as a safety measure to indicate any potential leakage of CO gas.

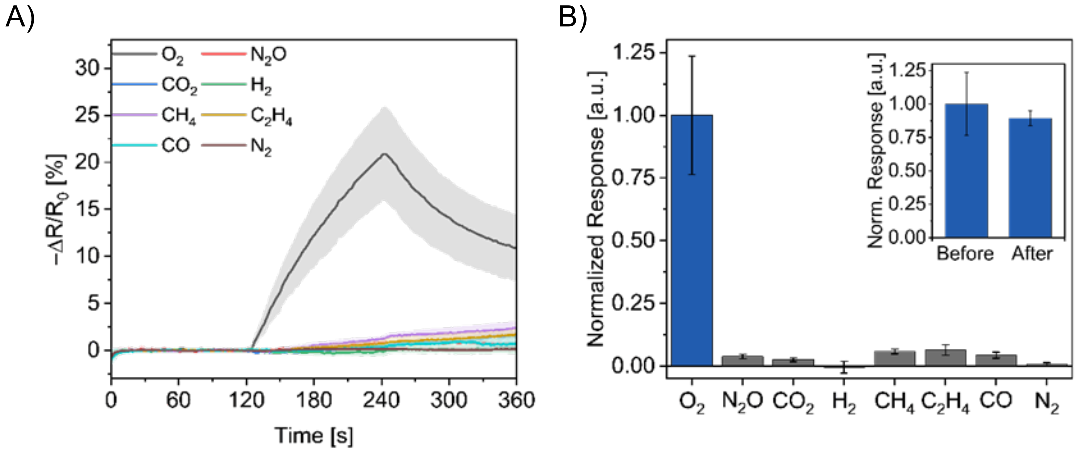

Figure S33. A) Normalized and drift-corrected resistance traces for 2 min exposure of **SWCNT-TiO$_2$-Re** to 1000 ppm O_2, N_2O, CO_2, H_2, CH_4, C_2H_4, CO, and N_2. B) Data represented as bar plot. The response of **SWCNT-TiO$_2$-Re** to 1000 ppm O_2 before and after the selectivity study is shown in the inset as bar plot. Shaded areas and error bars represent standard deviations (*N* = 4 chemiresistors); all data were collected under green light irradiation at room temperature using dry nitrogen carrier gas (R.H. = 0%).

Stability Studies

Device Storage

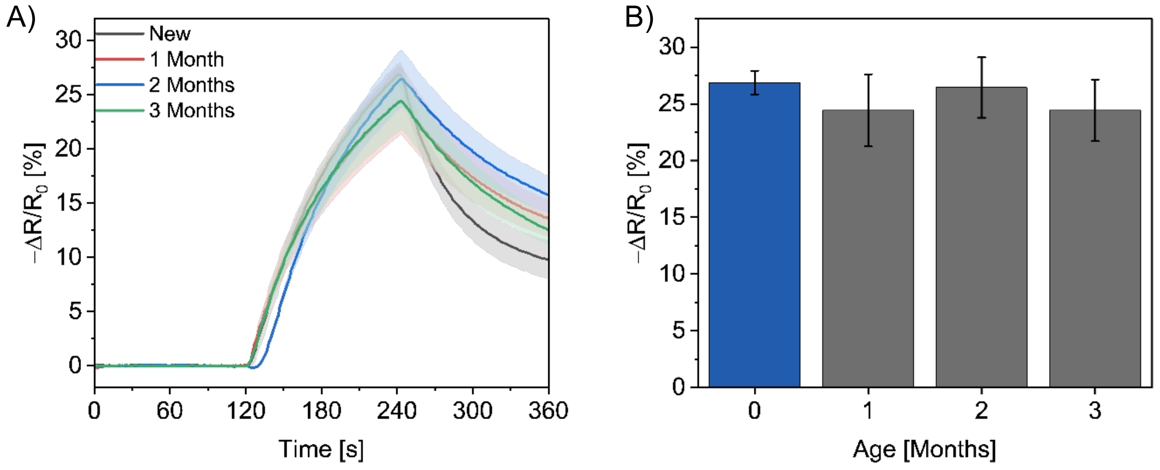

Figure S34. A) Normalized and drift-corrected resistance traces for 2 min exposure of **SWCNT-TiO2-Re** to 1000 ppm of O_2 after storage of the devices for 0, 1, 2 and 3 months, respectively. B) Data illustrated as bar plot. Shaded areas and error bars represent standard deviations (N = 4 chemiresistors); all data were collected under green light irradiation at room temperature using dry nitrogen carrier gas (R.H. = 0%).

Repeated O_2 Exposures

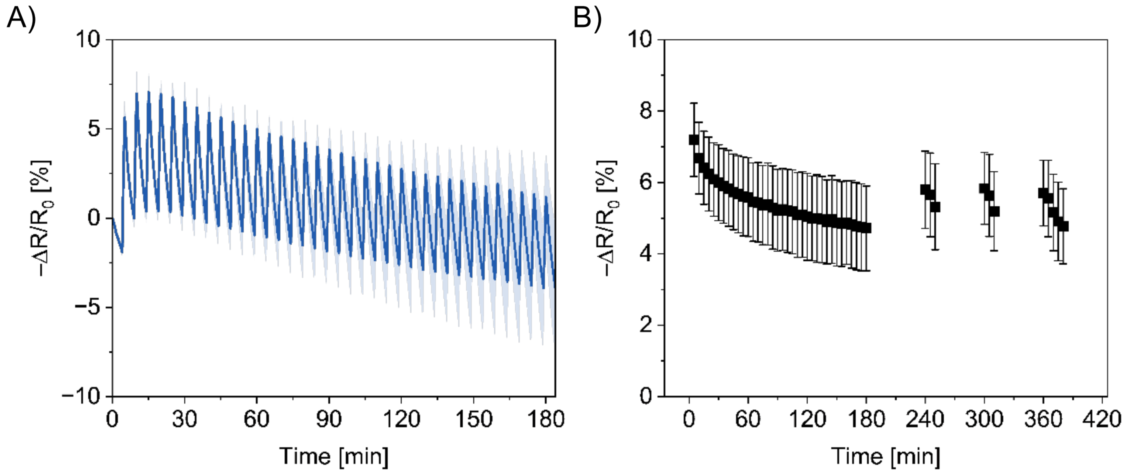

Figure S35. A) Normalized resistance trace for repeated 1 min exposures of **SWCNT-TiO2-Re** to 1000 ppm of O_2 for 180 min. B) Data illustrated as scatter plot with additional data points after 4 h, 5 h, and 6 h, respectively. Shaded areas and error bars represent standard deviations (N = 4 chemiresistors); all data were collected under green light irradiation at room temperature using dry nitrogen carrier gas (R.H. = 0%).

VI. O_2 Chemiresistor Comparison Table

Table S2. Comparison of different chemiresistive oxygen sensors according to various sensing performance metrics.

Entry	Material	Temperature	Irradiation	Sensitivity	$[O_2]$ Range Tested	Selectivity	Stability	Humidity
1	**SWCNT-TiO_2-Re**	r.t.	visible light	LOD = 157 ppb	20–200 000 ppm	H_2, CO_2, N_2O, CO, CH_4, C_2H_4, N_2	3 months	0–80% R.H. tolerated
2[12]	TiO_2/GaN Nanowires	r.t.	UV light	LOD = 0.45%	1–25%	N_2, CO_2, CH_4, H_2S, H_2	1 month	0–70% R.H. tolerated
3[13]	MWCNT	r.t.	no	3.6% for 100% O_2	0.3–100%	n.a.	n.a.	n.a.
4[14]	VO_x/TiO_2 Nanoflakelets	150–500 °C	no	35% for 100 ppm	0.01–4%	NO_2, H_2, NH_3	6 months	0–90% R.H. tolerated
5[15]	2D Bi_2O_2Se Nanoplatelets	r.t.	no	LOD < 0.25 ppm	up to 400 ppm	CO_2, CH_4, H_2, Ar	>1 month	n.a.
6[16]	Eu_8-SWCNT	r.t.	UV light	LOD = 0.8%	5–27%	CO_2, NH_3[c]	1 week	0–43% R.H. tolerated
7[17]	MWCNT-TiO_2	350–550 °C	no	LOD < 10 ppm	10 ppm	n.a.	n.a.	n.a.
8[18]	Pt-In_2O_3 Nanowires	50–300 °C	no	1.72 for 100 ppm	10–400 ppm	n.a.	n.a.	n.a.
9[19]	LaOCl–SnO_2 Hollow Spheres	r.t.	UV light	2.25 for 250 ppm	100–5000 ppm	H_2, CH_4, NH_3, CO_2	2 months	0–50% R.H. tolerated
10[20]	P-doped TiO_2	116 °C	no	29.6% for 100 ppm	100–1000 ppm	CO, H_2, NH_3, CO_2, EtOH	n.a.	0–50% R.H. tolerated
11[21]	$SrTi_{0.65}Fe_{0.35}O_{3-\delta}$ Nanofibers	600–950 °C	no	4.08 for 20% O_2	1–20%	n.a.	n.a.	n.a.
12[22]	Carbon Nitride/rGO	r.t.	UV light	LOD = 6 ppm[d]	300–100 000 ppm	H_2, CO_2	n.a.	reported at 100% R.H.

[c] The sensor responds to NO_2, which the authors accounted for using a sensor array.
[d] LOQ = 20 ppm was indicated by the authors. LOQ is the oxygen concentration that exceeds 10 times the standard deviation of the blank.

VII. Energy Levels of [(^{P}bpy)(CO)$_3$ReBr]

To estimate the excited-state potential of [(^{P}bpy)(CO)$_3$ReBr] that is relevant to electron injection into SWCNT-TiO_2 under light irradiation, a combination of electrochemical and spectroscopic data were utilized according to established literature procedures.[23] First, the ground-state potential of [(^{P}bpy)(CO)$_3$ReBr] [E(S/S$^+$)] was determined by cyclic voltammetry (Figure S36). The stored excitation energy of [(^{P}bpy)(CO)$_3$ReBr] was taken as the energy of the 0–0 transition between the lowest vibrational levels in the ground and excited states [$\Delta E_{0\text{–}0}$(S/S*)], determined from the intersection of the emission and absorption spectra of [(^{P}bpy)(CO)$_3$ReBr] (Figure S37).[24] With these values in hand, the excited-state potential of [(^{P}bpy)(CO)$_3$ReBr] [E(S*/S$^+$)] can be calculated according to:

$$E(\mathrm{S^*/S^+}) = E(\mathrm{S/S^+}) + \Delta E_{0\text{–}0}(\mathrm{S/S^*})$$

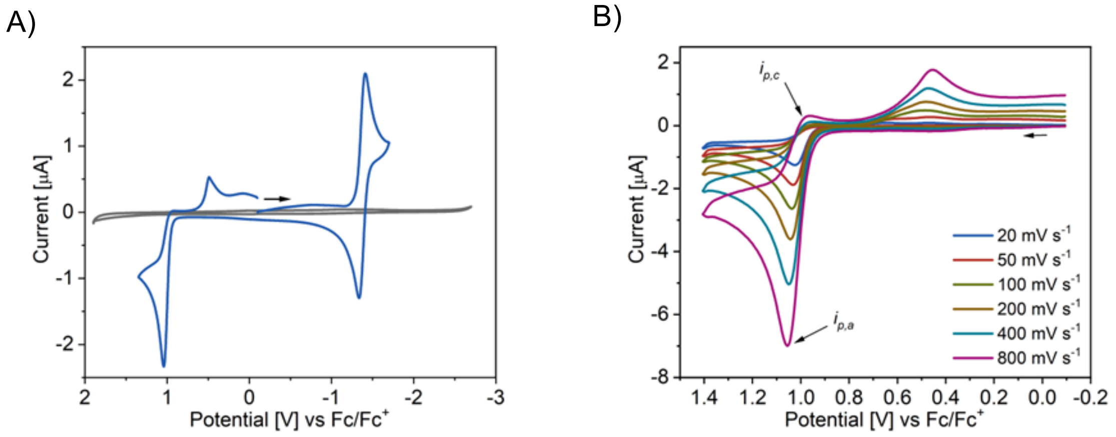

Figure S36. A) Cyclic voltammogram of [(^{P}bpy)(CO)$_3$ReBr] with 0.10 M [(n-Bu)$_4$(PF_6)] as supporting electrolyte measured at a scan rate of 100 mV/s in MeCN. B) Scan rate study of [(^{P}bpy)(CO)$_3$ReBr] to investigate the oxidative feature. A small reduction feature ($i_{p,c}$) starts appearing at higher scan rates that was used to determine the half-wave potential.

Note. Owing to the irreversibility of the anodic feature at low and intermediate scan rates, E(S/S$^+$) was calculated as an average of the inflection points leading to the anodic peak potential ($i_{p,a}$) at various scan rates (+1.00 V vs Fc/Fc$^+$; +1.63 V vs NHE).[25] At higher scan rates, the corresponding cathodic feature began to appear ($i_{p,c}$, Figure S36B). Calculating E(S/S$^+$) as the average of the oxidative and reductive peak potentials (i.e. as a half-wave potential) afforded the same value as that obtained by the inflection point method.[26]

Therefore, it follows that E(S*/S$^+$) = −0.93 V vs NHE for [(^{P}bpy)(CO)$_3$ReBr]. This value is comparable with a previous report for the related complex [(deeb)(CO)$_3$ReBr] (deeb = 4,4'-(C(O)OEt)$_2$-2,2'-bipyridine), where E(S*/S$^+$) was found to be –0.76 V vs NHE.[27]

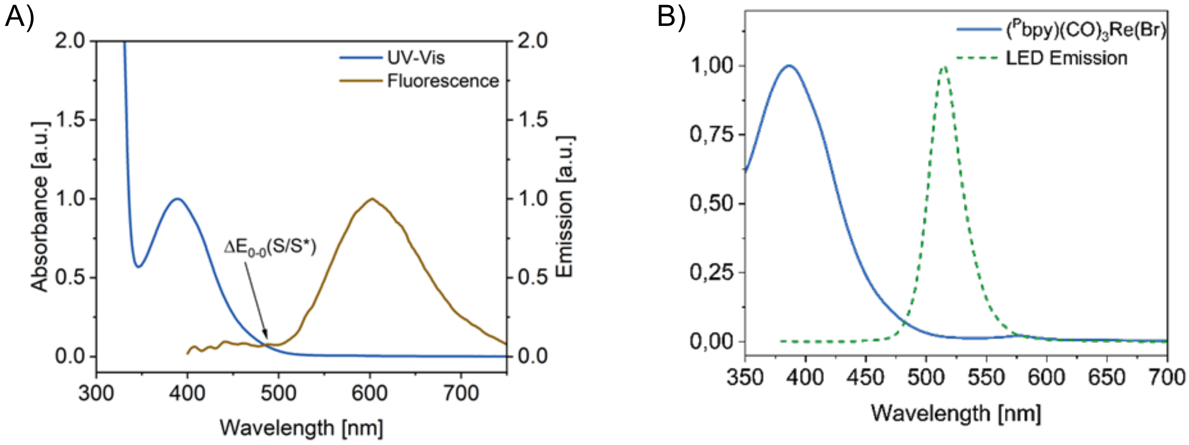

Figure S37. A) Solution-state UV-vis (blue) and fluorescence (gold) spectra of [(^{P}bpy)(CO)$_3$ReBr] measured in DMF. The UV-vis and fluorescence spectra were normalized to the MLCT peak at 389 nm and to the emission peak at 602 nm, respectively. The emission data was smoothened using the Savitzky–Golay method. The value ΔE_{0-0}(S/S*) was determined from the intersection of the spectra (484 nm = 2.56 eV). B) Spectrum showing the overlap of the normalized absorbance of [(^{P}bpy)(CO)$_3$ReBr] and the LED emission.

VIII. Synthetic Protocols

Synthesis of Tetraethyl [2,2'-Bipyridine]-4,4'-diylbis(phosphonate)

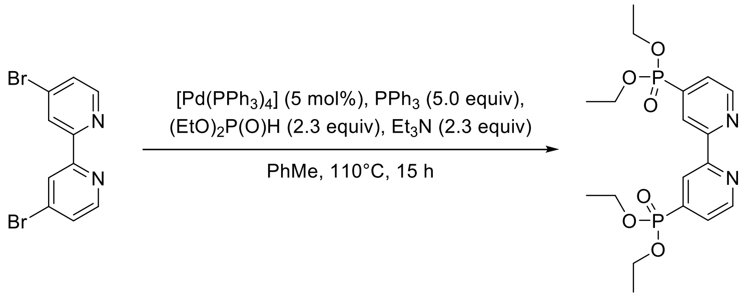

The synthesis was adapted from a literature report.[28] In an oven-dried 250 mL two-neck round-bottom flask equipped with a stirring bar, 4,4'-dibromo-2,2'-bipyridine (2.00 g, 6.37 mmol, 1.0 equiv), PPh_3 (8.35 g, 31.9 mmol, 5.0 equiv), diethyl phosphite (1.90 mL, 14.7 mmol, 2.3 equiv), $[Pd(PPh_3)_4]$ (0.37 mg, 0.32 mmol, 0.05 equiv), and Et_3N (2.00 mL, 14.7 mmol, 2.3 equiv) were refluxed in dry toluene (60 mL) at 110 °C for 15 h under an argon atmosphere. After cooling to room temperature, the yellow reaction mixture was extracted with aqueous ammonia. The colorless organic phase was washed with water and brine, then dried over $MgSO_4$, filtered through Celite, and concentrated under reduced pressure. The crude product was purified by silica gel flash column chromatography (0–4% MeOH in CH_2Cl_2). PPh_3 eluted at 100% CH_2Cl_2. The product partly coeluted with triphenyl phosphine oxide. Pure fractions were selected by TLC spotting and afforded the product as a white solid (1.20 g, 44%).

^{1}H NMR (300 MHz, $CDCl_3$, 298 K): δ 8.89 – 8.78 (m, 2H), 8.74 (d, $^3J_{HP}$ = 14.2 Hz, 2H), 7.92 (ddd, $^3J_{HP}$ = 13.0 Hz, $^3J_{HH}$ = 4.8 Hz, $^4J_{HP}$ = 1.5 Hz, 2H), 4.29 – 4.04 (m, 8H), 1.333 (t, $^3J_{HH}$ = 7.1 Hz, 12 H). **^{31}P{^{1}H} NMR** (121 MHz, $CDCl_3$, 298 K): δ 14.75.

Synthesis of [(^{P}bpy)(CO)$_3$ReBr]

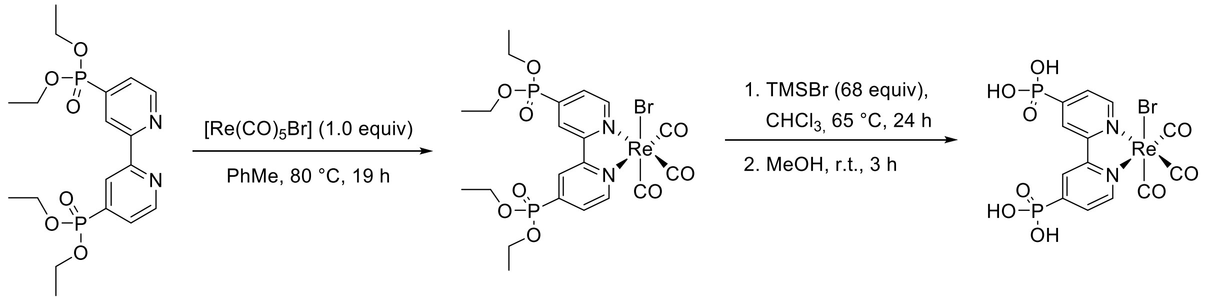

The synthesis was adapted from a literature report.[29] In the glovebox, tetraethyl [2,2'-bipyridine]-4,4'-diylbis(phosphonate) (200 mg, 0.47 mmol, 1.00 equiv) and $[Re(CO)_5Br]$ (191 mg, 0.47mmol, 1.00 equiv) were combined in an oven-dried microwave vial equipped with a stirring bar and dissolved in anhydrous toluene (50 mL). The reaction mixture was heated to 80 °C for 19 h in a microwave reactor. The solvent was then evaporated under reduced pressure. Precipitation from CH_2Cl_2/hexane afforded the intermediate product the as an orange solid (340 mg, 94%). The intermediate (300 mg, 0.39 mmol) was then dried overnight *in vacuo* in a two-neck 50 mL round-bottom flask equipped with a stirring bar and a reflux condenser. Under an argon atmosphere, anhydrous $CHCl_3$ (15 mL) and bromotrimethylsilane (3.5 mL, 26.7 mmol, 68.0 equiv) were added. The reaction mixture was refluxed at 65 °C for 24 h. The volatiles were evaporated *in vacuo* after cooling to room temperature. Dry MeOH (3 mL) was added to the residue, the mixture was stirred for 3 h and

then dried *in vacuo*. The crude was purified by precipitation from MeOH/Et_2O affording [(^{P}bpy)$(CO)_3$ReBr] as a bright orange solid (166 mg, 65%).

^{1}H NMR (300 MHz, DMSO-d_6, 298 K): δ 9.17 (dd, $^3J_{HH}$ = 5.5 Hz, $^4J_{HP}$ = 3.5 Hz, 2H), 8.75 (d, $^3J_{HP}$ = 13.1 Hz, 2H), 7.92 (dd, $^3J_{HH}$ = 5.5, $^3J_{HP}$ = 12.1 Hz, 2H). **^{13}C{^{1}H} NMR** (126 MHz, DMSO-d_6, 298 K): δ 197.52 (s, CO), 189.34 (s, CO), 155.12 (d, $^3J_{CP}$ = 13.5 Hz), 153.93 (d, $^3J_{CP}$ = 11.8 Hz), 147.62 (d, $^1J_{CP}$ = 173.6 Hz), 128.96 (d, $^2J_{CP}$ = 8.0 Hz), 125.07 (d, $^2J_{CP}$ = 9.3 Hz). **^{31}P{^{1}H} NMR** (202 MHz, DMSO-d_6, 298 K): δ 5.56.

IR (KBr) $\tilde{\nu}_{max}$ [cm^{-1}]: 2033 (CO), 1915 (CO).

UV-vis (DMF): λ_{max} 301 nm ($\varepsilon_{max} = 1.3 \cdot 10^4$ M^{-1} cm^{-1}), λ_{MLCT} 389 nm ($\varepsilon_{MLCT} = 3.3 \cdot 10^3$ M^{-1} cm^{-1}).

Spectra

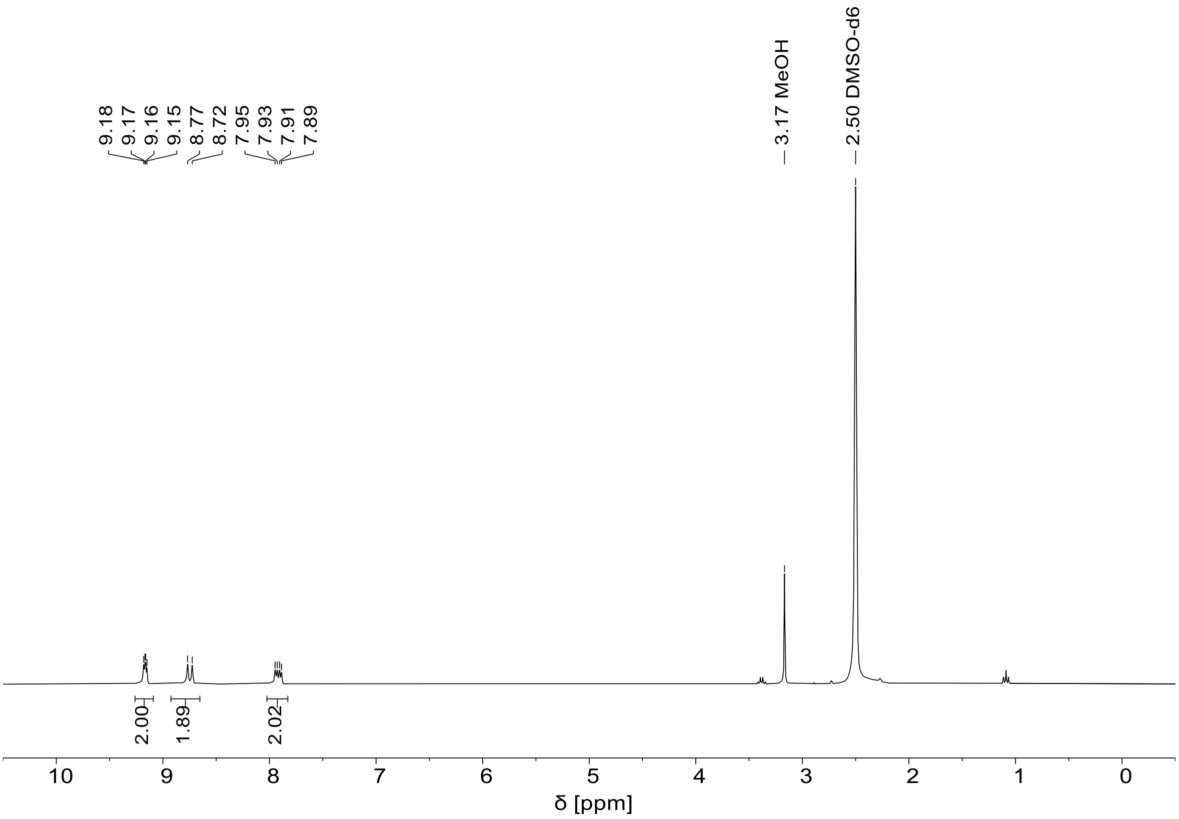

Figure S38. ^{1}H NMR (300 MHz, DMSO-d_6, 298 K) spectrum of [(^{P}bpy)(CO)$_3$ReBr].

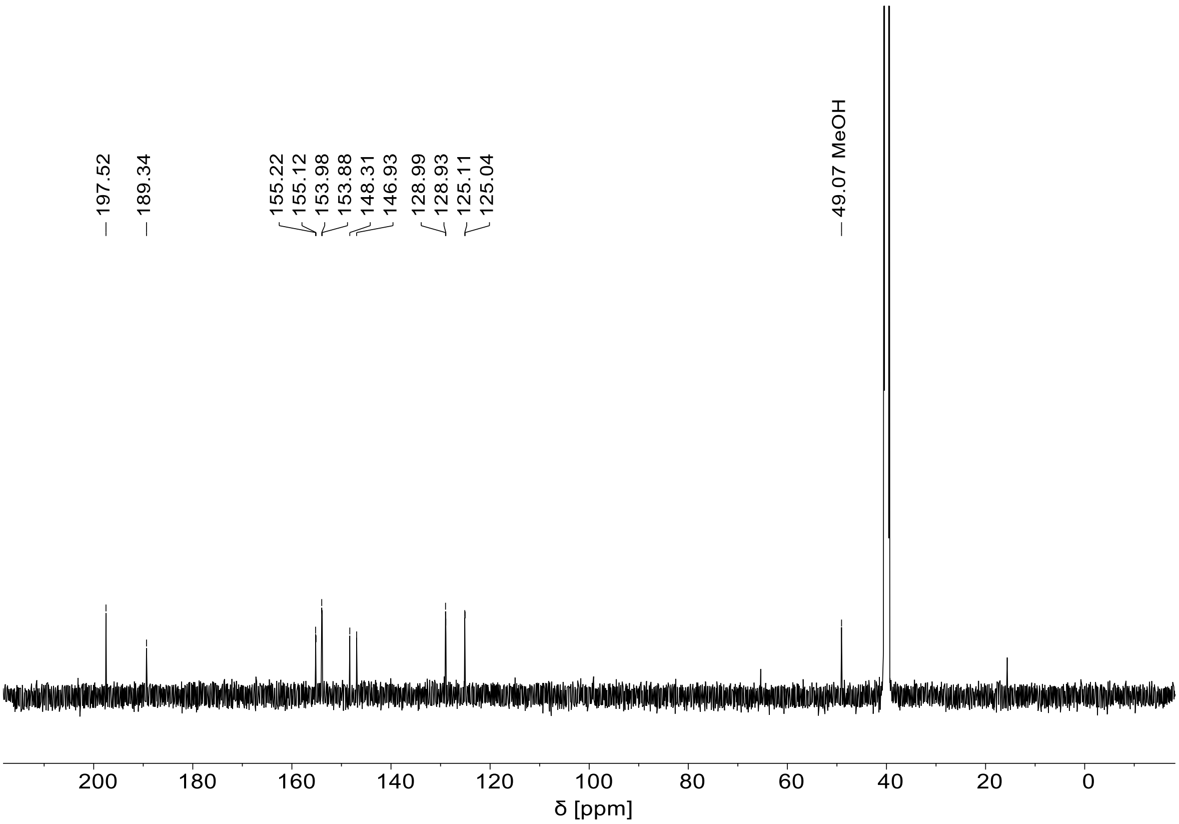

Figure S39. ^{13}C{^{1}H} NMR (126 MHz, DMSO-d_6, 298 K) spectrum of [(^{P}bpy)(CO)$_3$ReBr].

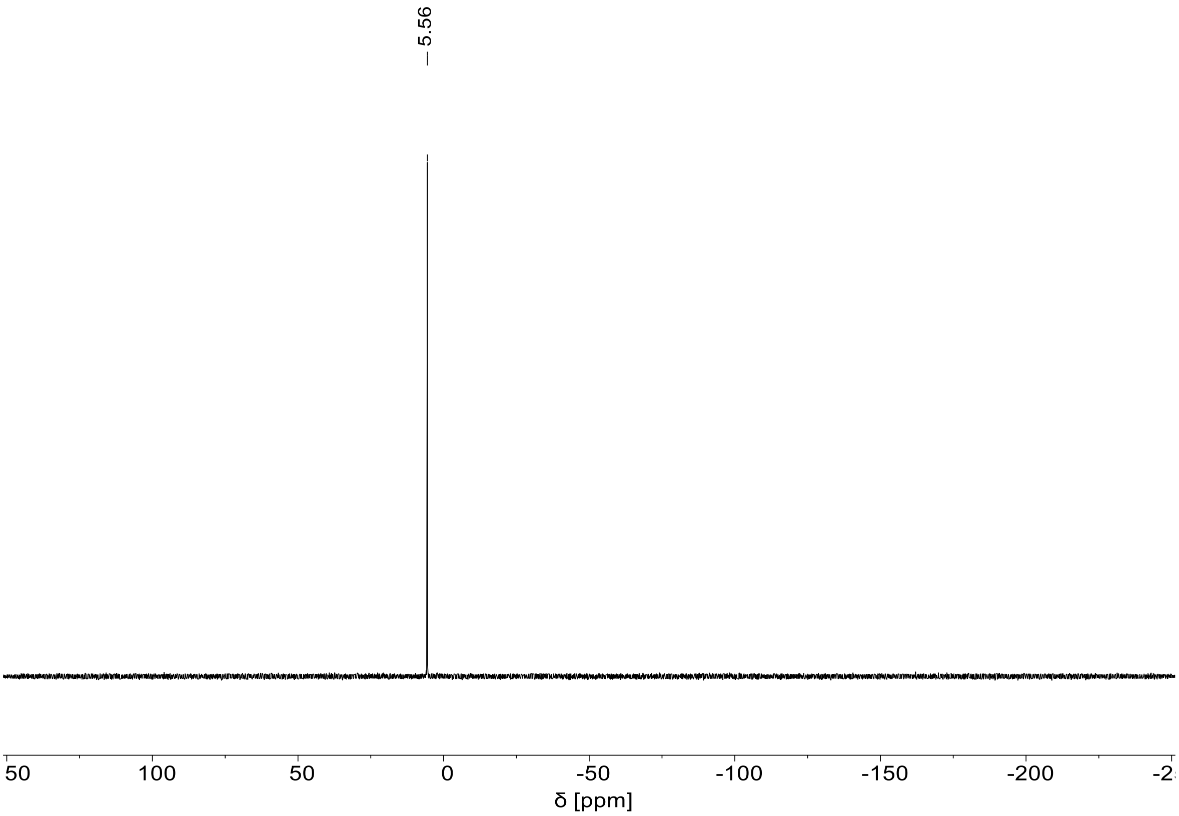

Figure S40. $^{31}P\{^{1}H\}$ NMR (202 MHz, DMSO-d_6, 298 K) spectrum of [(^{P}bpy)(CO)$_3$ReBr].

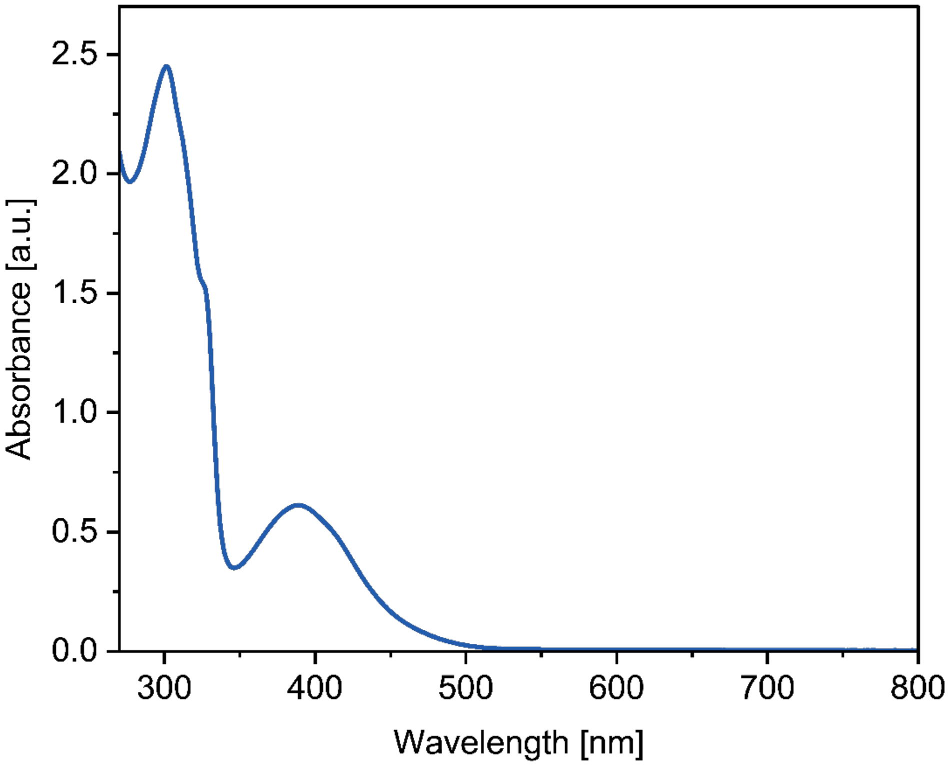

Figure S41. UV-vis (DMF) spectrum of [(^{P}bpy)(CO)$_3$ReBr].

IX. References

1 N. Fairley, V. Fernandez, M. Richard-Plouet, C. Guillot-Deudon, J. Walton, E. Smith, D. Flahaut, M. Greiner, M. Biesinger, S. Tougaard, D. Morgan, J. Baltrusaitis, *Appl. Surf. Sci. Adv.* **2021**, *5*, 100112.

2 M. Ding, D. C. Sorescu, A. Star, *J. Am. Chem. Soc.* **2013**, *135*, 9015.

3 P. Makuła, M. Pacia, W. Macyk, *J. Phys. Chem. Lett.* **2018**, *9*, 6814.

4 A. Bhogi, B. Srinivas, M. Shareefuddin, P. Kistaiah, *Mater. Today: Proc.* **2023**, *92*, 727.

5 D. Souri, Z. E. Tahan, *Appl. Phys. B* **2015**, *119*, 273.

6 A. Bhogi, B. Srinivas, P. Padmavathi, K. Venkataramana, K. K. Ganta, M. Shareefuddin, P. Kistaiah, *Opt. Mater.* **2022**, *133*, 112911.

7 A. E. R. Friedmann, K. Thiel, T. M. Gesing, P. Plagemann, *Surf. Coat. Technol.* **2018**, *344*, 710721.

8 R. López, R. Gómez, J. *Sol-Gel Sci. Technol.* **2012**, *61*, 1.

9 E. S. Welter, S. Garg, R. Gläser, M. Goepel, *ChemPhotoChem* **2023**, *7*, e202300001.

10 S.-X. L. Luo, T. M. Swager, *Nat. Rev. Methods Primers* **2023**, *3*, 73.

11 L. A. Currie, *Pure Appl. Chem.* **1995**, *67*, 1699.

12 Z. Li, H. Huang, D. Zhao, S. Chen, W. Cai, T. Tang, *Sens. Actuators B Chem.* **2024**, *401*, 135015.

13 K. Rajavel, M. Lalitha, J. K. Radhakrishnan, L. Senthilkumar, R. T. R. Kumar, *ACS Appl. Mater. Interfaces* **2015**, *7*, 23857.

14 A. V. Raghu, K. K. Karuppanan, B. Pullithadathil, *ACS Sens.* **2018**, *3*, 1811.

15 S. Xu, H. Fu, Y. Tian, T. Deng, J. Cai, J. Wu, T. Tu, T. Li, C. Tan, Y. Liang, C. Zhang, Z. Liu, Z. Liu, Y. Chen, Y. Jiang, B. Yan, H. Peng, *Angew. Chem. Int. Ed.* **2020**, *59*, 17938.

16 D. R. Kauffman, C. M. Shade, H. Uh, S. Petoud, A. Star, *Nat. Chem.* **2009**, *1*, 500.

17 E. Llobet, E. H. Espinosa, E. Sotter, R. Ionescu, X. Vilanova, J. Torres, A. Felten, J. J. Pireaux, X. Ke, G. V. Tendeloo, F. Renaux, Y. Paint, M. Hecq, C. Bittencourt, *Nanotechnology* **2008**, *19*, 375501.

18 S. S. Kim, J. Y. Park, S.-W. Choi, H. S. Kim, H. G. Na, J. C. Yang, H. W. Kim, *Nanotechnology* **2010**, *21*, 415502.

19 Y. Xiong, W. Lu, D. Ding, L. Zhu, X. Li, C. Ling, Q. Xue, *ACS Sens.* **2017**, *2*, 679.

20 Z. Han, J. Wang, L. Liao, H. Pan, S. Shen, J. Chen, *Appl. Surf. Sci.* **2013**, *273*, 349.

21 S.-H. Choi, S.-J. Choi, B. K. Min, W. Y. Lee, J. S. Park, I.-D. Kim, *Macromol. Mater. Eng*. **2013**, *298*, 521.

22 J. E. Ellis, D. C. Sorescu, S. C. Burkert, D. L. White, A. Star, *ACS Appl. Mater. Interfaces* **2017**, *9*, 27142.

23 A. Hagfeldt, G. Boschloo, L. Sun, L. Kloo, H. Pettersson, *Chem. Rev.* **2010**, *110*, 6595.

24 D. Kim, T. S. Teets, *Chem. Phys. Rev.* **2022**, *3*, 021302.

25 E. M. Espinoza, J. A. Clark, J. Soliman, J. B. Derr, M. Morales, V. I. Vullev, *J. Electrochem. Soc.* **2019**, *166*, H3175.

26 C. M. Cardona, W. Li, A. E. Kaifer, D. Stockdale, G. C. Bazan, *Adv. Mater.* **2011**, *23*, 2367.

27 G. M. Hasselmann, G. J. Meyer, *J. Phys. Chem. B* **1999**, *103*, 7671.

28 V. Penicaud, F. Odobel, B. Bujoli, *Tetrahedron Lett.* **1998**, *39*, 3689.

29 C. D. Windle, E. Pastor, A. Reynal, A. C. Whitwood, Y. Vaynzof, J. R. Durrant, R. N. Perutz, E. Reisner, *Chem. Eur. J.* **2015**, *21*, 3746.
